# Diagnostic and prognostic value of hematological and immunological markers in COVID-19 infection: A meta-analysis of 6320 patients

**DOI:** 10.1371/journal.pone.0238160

**Published:** 2020-08-21

**Authors:** Rami M. Elshazli, Eman A. Toraih, Abdelaziz Elgaml, Mohammed El-Mowafy, Mohamed El-Mesery, Mohamed N. Amin, Mohammad H. Hussein, Mary T. Killackey, Manal S. Fawzy, Emad Kandil

**Affiliations:** 1 Department of Biochemistry and Molecular Genetics, Faculty of Physical Therapy, Horus University - Egypt, New Damietta, Egypt; 2 Department of Surgery, Tulane University, School of Medicine, New Orleans, Louisiana, United States of America; 3 Genetics Unit, Department of Histology and Cell Biology, Faculty of Medicine, Suez Canal University, Ismailia, Egypt; 4 Department of Microbiology and Immunology, Faculty of Pharmacy, Mansoura University, Mansoura, Egypt; 5 Department of Microbiology and Immunology, Faculty of Pharmacy, Horus University - Egypt, New Damietta, Egypt; 6 Department of Biochemistry, Faculty of Pharmacy, Mansoura University, Mansoura, Egypt; 7 Department of Medical Biochemistry and Molecular Biology, Faculty of Medicine, Suez Canal University, Ismailia, Egypt; 8 Department of Biochemistry, Faculty of Medicine, Northern Border University, Arar, KSA; 9 Division of Endocrine and Oncologic Surgery, Department of Surgery, Tulane University, School of Medicine, New Orleans, Los Angeles, United States of America; Taibah University, SAUDI ARABIA

## Abstract

**Objective:**

Evidence-based characterization of the diagnostic and prognostic value of the hematological and immunological markers related to the epidemic of Coronavirus Disease 2019 (COVID-19) is critical to understand the clinical course of the infection and to assess in development and validation of biomarkers.

**Methods:**

Based on systematic search in Web of Science, PubMed, Scopus, and Science Direct up to April 22, 2020, a total of 52 eligible articles with 6,320 laboratory-confirmed COVID-19 cohorts were included. Pairwise comparison between severe *versus* mild disease, Intensive Care Unit (ICU) *versus* general ward admission and expired *versus* survivors were performed for 36 laboratory parameters. The pooled standardized mean difference (SMD) and 95% confidence intervals (CI) were calculated using the DerSimonian Laird method/random effects model and converted to the Odds ratio (OR). The decision tree algorithm was employed to identify the key risk factor(s) attributed to severe COVID-19 disease.

**Results:**

Cohorts with elevated levels of white blood cells (WBCs) (OR = 1.75), neutrophil count (OR = 2.62), D-dimer (OR = 3.97), prolonged prothrombin time (PT) (OR = 1.82), fibrinogen (OR = 3.14), erythrocyte sedimentation rate (OR = 1.60), procalcitonin (OR = 4.76), IL-6 (OR = 2.10), and IL-10 (OR = 4.93) had higher odds of progression to severe phenotype. Decision tree model (sensitivity = 100%, specificity = 81%) showed the high performance of neutrophil count at a cut-off value of more than 3.74x10^9^/L for identifying patients at high risk of severe COVID‐19. Likewise, ICU admission was associated with higher levels of WBCs (OR = 5.21), neutrophils (OR = 6.25), D-dimer (OR = 4.19), and prolonged PT (OR = 2.18). Patients with high IL-6 (OR = 13.87), CRP (OR = 7.09), D-dimer (OR = 6.36), and neutrophils (OR = 6.25) had the highest likelihood of mortality.

**Conclusions:**

Several hematological and immunological markers, in particular neutrophilic count, could be helpful to be included within the routine panel for COVID-19 infection evaluation to ensure risk stratification and effective management.

## Introduction

Coronavirus disease– 2019 (COVID-19) is a disease that was detected in December 2019 in Wuhan, China, and led to the risk of mortality of about 2% [1]. This disease is caused due to infection with a recently arising zoonotic virus known as the Severe Acute Respiratory Syndrome Coronavirus 2 (SARS-CoV-2) [2]. Previously, infection with coronaviruses appeared in 2002 within China in the form of SARS-CoV, and it appeared later also in 2012 within Saudi Arabia that was known as Middle East Respiratory Syndrome (MERS-CoV) [3, 4]. All these coronaviruses are enveloped positive-strand RNA viruses that are isolated from bats that can be transferred from animals to humans, human to human, and animals to animals [5]. They share a similarity in the clinical symptoms in addition to specific differences that have been recently observed [5–7]. The symptoms of this disease appear with different degrees that start in the first seven days with mild symptoms such as fever, cough, shortness of breath, and fatigue [8]. Afterward, critical symptoms may develop in some patients involving dyspnea and pneumonia that require patient’s management in intensive care units to avoid the serious respiratory complications that may lead to death [9]. However, there are no specific symptoms to diagnose coronavirus infection, and accurate testing depends on the detection of the viral genome using the reverse transcription-polymerase chain reaction (RT-PCR) analysis [10].

Unfortunately, COVID-19 is not limited to its country of origin, but it has spread all over the world. Therefore, there is no wonder emerging research has been directed to provide information and clinical data of patients infected with this virus that may help to not only to the early detection in different patient categories, but it will also help in the characterization of the viral complications with other chronic diseases [1, 2, 6, 9]. However, there is no sufficient data that characterize the changes in the hematological and immunological parameters in COVID-19 patients. In the current comprehensive meta-analysis study, we aimed to analyze different hematological, inflammatory, and immunological markers in COVID-19 patients at different clinical stages in different countries that may help in the early detection of COVID-19 infection and to discriminate between severity status of the disease to decrease the death risk.

## Materials and methods

### Search strategy

This current meta-analysis was carried out according to the Preferred Reporting Items for Systematic reviews and Meta-analysis (PRISMA) statement [11] (S1 Table). Relevant literature was retrieved from Web of Science, PubMed, Scopus, and Science Direct search engines up to April 22, 2020. Our search strategy included the following terms: “Novel coronavirus 2019”, “2019 nCoV”, “COVID-19”, “Wuhan coronavirus,” “Wuhan pneumonia,” or “SARS-CoV-2”. Besides, we manually screened out the relevant potential article in the references selected. The above process was performed independently by three participants.

### Study selection

No time or language restriction was applied. Inclusion criteria were as follows: (1) Types of Studies: retrospective, prospective, observational, descriptive or case control studies reporting laboratory features of COVID-19 patients; (2) Subjects: diagnosed patients with COVID-19 (3) Exposure intervention: COVID-19 patients diagnosed with Real Time-Polymerase Chain Reaction, radiological imaging, or both; with hematological testing included: complete blood picture (white blood cells, neutrophil count, lymphocyte count, monocyte count, eosinophils count, basophils, red blood cells, hemoglobin, hematocrit, and platelet count), coagulation profile (prothrombin time, international normalized ratio, activated partial thromboplastin time, thrombin time, fibrinogen, and D-dimer) or immunological parameters including inflammatory markers (ferritin, erythrocyte sedimentation rate, procalcitonin, and C-reactive protein), immunoglobulins (IgA, IgG, and IgM), complement tests (C3 and C4), interleukins (IL-4, IL-6, IL-8, IL-10, IL-2R, and TNF-α), and immune cells (B lymphocytes, T lymphocytes, CD4^+^ T cells, and CD8^+^ T cells); and (4) Outcome indicator: the mean and standard deviation or median and interquartile range for each laboratory test. The following exclusion criteria were considered: (1) Case reports, reviews, editorial materials, conference abstracts, summaries of discussions, (2) Insufficient reported data information; or (3) *In vitro* or *in vivo* studies.

### Data abstraction

Four investigators separately conducted literature screening, data extraction, and literature quality evaluation, and any differences were resolved through another two reviewers. Information extracted from eligible articles in a predesigned form in excel, including the last name of the first author, date and year of publication, journal name, study design, country of the population, sample size, mean age, sex, and quality assessment.

### Quality assessment

A modified version of the Newcastle-Ottawa scale (NOS) was adopted to evaluate the process in terms of queue selection, comparability of queues, and evaluation of results [12, 13]. The quality of the included studies was assessed independently by three reviewers, and disagreements were resolved by the process described above. Higher NOS scores showed a higher literature quality. NOS scores of at least six were considered high-quality literature.

### Statistical analysis

All data analysis was performed using OpenMeta[Analyst] [14] and comprehensive meta-analysis software version 3.0 [15]. First, a single-arm meta‐analysis for laboratory tests was performed. The standardized mean difference (SMD) and 95%confidence intervals (CI) were used to estimate pooled results from studies. Medians and interquartile range were converted to mean and standard deviation (SD) using the following formulas: [Mean = (Q1+median+Q3)/3] and [SD = IQR/1.35], whereas, values reported in the articles as mean and 95%CI were estimated using the following formula [SD = √N * (Upper limit of CI–Lower limit of CI)/3.92]. A continuous random-effect model was applied using the DerSimonian-Laird (inverse variance) method [16, 17].

Next, in the presence of individual patient data, single-armed observed values were converted to two-armed data to act as each other’s control group based on covariate information. Only studies investigating different outcomes were considered as potential matched pairs, and two-arm meta-analysis was applied to compare between mild *versus* severe COVID-19 infection (based on the results of the chest radiography, clinical examination, and symptoms), ICU admission *versus* general ward admission, and expired *versus* survivors. Meta-analysis for each outcome was processed using a random-effects model since heterogeneity among studies was expected. For pairwise comparison, estimates of SMD served as quantitative measures of the strength of evidence, which were then converted to the odds ratio (OR) with 95%CI for better interpretation by clinical domains.

### Decision tree to identify predictors for poor outcomes

Using laboratory features for clinical prediction, the decision tree algorithm was employed to identify the key risk factors attributed to severe COVID-19 infection, which include a count of studies ≥10. The accuracy of the model was measured by the Area Under the Receiver Operating Characteristic (ROC) Curve (AUC), which depicts the true positive rate versus the false positive rate at various discrimination thresholds. The markers that have the highest AUC were identified, and the sensitivity and specificity of the cut-off threshold level were determined. R Studio was employed using the following packages: *tidyverse*, *magrittr*, *rpart*, *caret*, and *pROC*.

### Trial sequential analysis (TSA)

The statistical trustworthiness of this meta-analysis assessment was conducted using TSA through combining the cumulative sample sizes of all appropriate records with the threshold of statistical impact to diminish the accidental errors and enhance the intensity of expectations [18]. Two side trials with “type I error (α)” along with power set at 5% and 80% were employed. In the case of the “Z-curve” traverses the TSA monitoring boundaries, a reasonable degree of impact was accomplished, and no supplementary trials are crucial. Nevertheless, in case of the “Z-curve” failed to achieve the boundary limits, the estimated information size has not accomplished the required threshold to attract appropriate decisions and advance trials are mandatory. TSA platform (version 0.9.5.10 beta) was operated in the experiment.

### Assessment of heterogeneity and publication bias

After that, the heterogeneity was evaluated using Cochran’s Q statistic and quantified by using I^2^ statistics, which represents an estimation of the total variation across studies beyond chance. Articles were considered to have significant heterogeneity between studies when the *p-value* less than 0.1 or I^2^ greater than 50%. Subgroup analysis was performed based on the study sample size (≤50 patients compared to >50 patients) and the origin of patients (Wuhan city versus others). Also, sensitivity analyses and meta-regression with the random-effects model using restricted maximum likelihood algorithm were conducted to explore potential sources of heterogeneity.

Finally, publication bias was assessed using a funnel plot and quantified using Begg’s and Mazumdar rank correlation with continuity correction and Egger’s linear regression tests. Asymmetry of the collected studies’ distribution by visual inspection or *P-value* < 0.1 indicated obvious publication bias [19]. The Duval and Tweedie’s trim and fill method’s assumption were considered to reduce the bias in pooled estimates [20].

## Results

### Literature search

**Fig 1:**
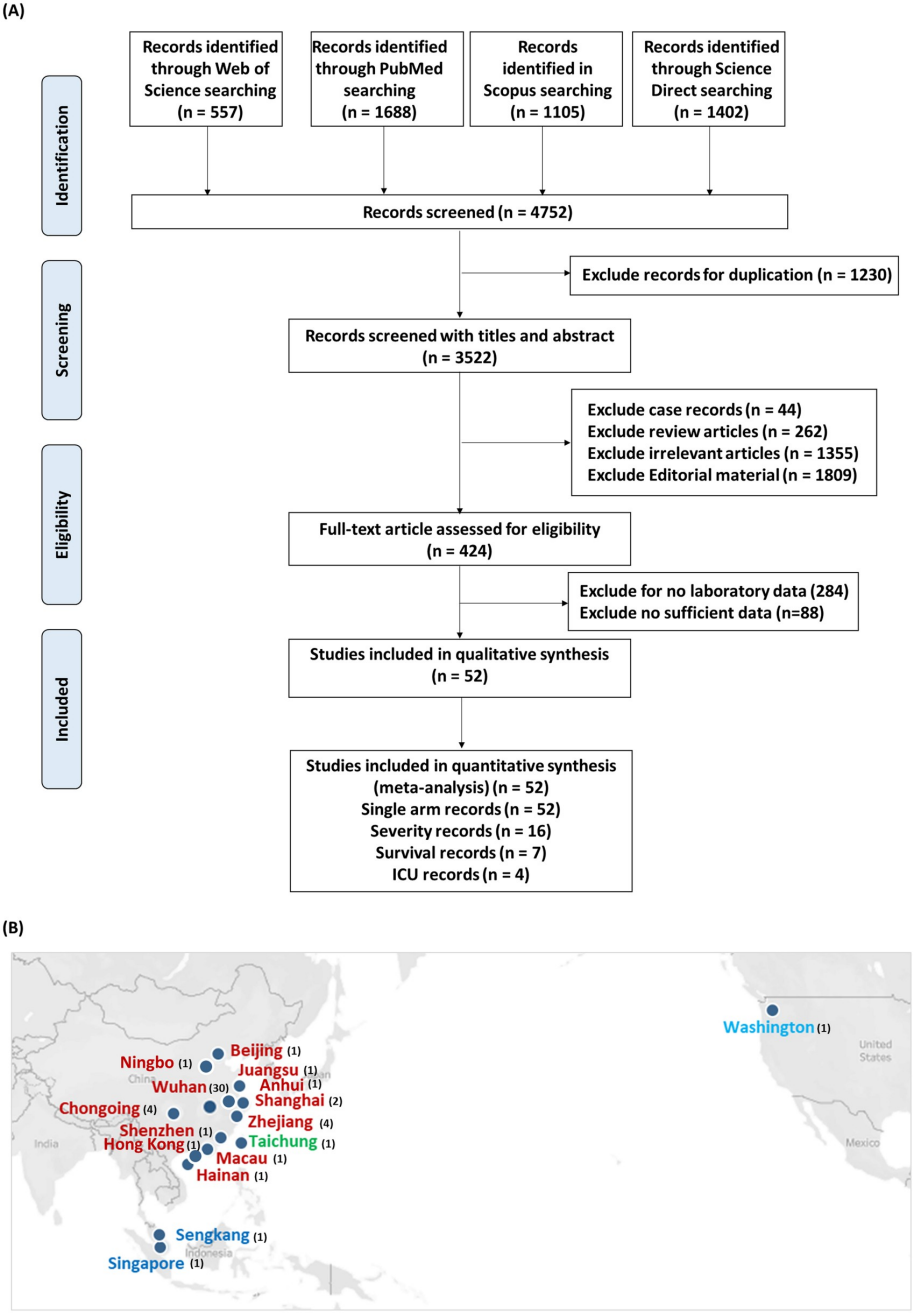
Literature search process. (A) Workflow for screening and selecting relevant articles. (B) Map showing the location of the studies. Studies conducted in China (red), Taiwan (green), Singapore (blue), and USA (light blue) are shown with the number of studies between brackets. Data source Tableau 2020.1 Desktop Professional Edition (https://www.tableau.com/).

A flowchart outlining the systematic review search results is described in Fig 1A. A total of 4752 records were identified through four major electronic databases till April 22, 2020 including Web of Science (n = 557), PubMed (n = 1688), Scopus (n = 1105) and Science Direct (n = 1402). Upon reviewing the retrieved articles, a total of 1230 records were excluded for duplication, and 3522 unique records were initially identified. Following screening of titles and abstracts, several studies were excluded for being case reports (n = 44), review articles (n = 262), irrelevant publications (n = 1355), or editorial materials (n = 1809). The resulted 424 full-text publications were further assessed for eligibility, during which 372 records were removed for lacking sufficient laboratory data. Ultimately, a total of 52 eligible articles were included for the quantitative synthesis of this meta-analysis study, with 52 records represented single-arm analysis [1, 9, 21–70], 16 records represented two-arms severity analysis [24, 26, 32, 34, 37, 40, 41, 45, 46, 50, 51, 63, 64, 66, 69, 70]; meanwhile, 7 and 4 records were utilized for survival [9, 30, 53, 55, 61, 67, 68] and ICU admission [1, 31, 36, 52] analyses, respectively.

#### Characteristics of the included studies

Our review included 52 studies that were published from January 24 through April 22, 2020, including 48 articles from China [Wuhan (30), Chongqing (4), Zhejiang (4), Shanghai (2), Ningbo (1), Hong Kong (1), Shenzhen (1), Anhui (1), Macau (1), Hainan (1), Jiangsu (1), and Beijing (1)], two articles from Singapore [Singapore and Sengkang], one article from Taiwan [Taichung], and one article from USA [Washington] (Fig 1B). The main characteristics of eligible studies are shown in Table 1. A total of 6320 patients with SARS‐CoV‐2 infection were enrolled across the articles. Most records (n = 47) were retrospective case studies, while other study design included two prospective cohort studies, one observational cohort study, one descriptive case series, and one case-control study. Our team stratified 36 different laboratory parameters into seven subclasses, including complete blood picture, coagulation profile, immunological markers, immunoglobulins, complement tests, interleukins, and immune cells, as previously described in the methodology. Regarding quality score assessment, 39 studies achieved a score higher than six out of a maximum of nine (high quality), while the remaining 13 studies earned a score equal or lower than six (low quality), as shown in Table 1.

**Table 1:** General characteristics of the included studies.

First Author	Publication* date (dd-mm)	Continent	Country	Study design	Sample size	Quality score	Mean age, years	Female %	Outcome	Ref.
Zhu Z	22-April	Ningbo	China	Retrospective case study	127	9	50.9 (15.3)	64.6%	Severity	[70]
Liu X	20-April	Wuhan	China	Retrospective case study	124	8	56 (12)	57.1%	Severity	[40]
Chen X	18-April	Wuhan	China	Retrospective case study	48	9	64.6 (18.1)	22.9%	Severity	[26]
Chen G	13-April	Wuhan	China	Retrospective case study	21	8	57 (11.1)	19%	Severity	[24]
He R	12-April	Wuhan	China	Retrospective case study	204	9	48.3 (20.7)	61.3%	Severity	[34]
Zhang G	09-April	Wuhan	China	Retrospective case study	221	9	53.5 (20.4)	51.1%	Severity	[63]
Lei S	04-April	Wuhan	China	Retrospective case study	34	9	53.7 (14.8)	58.8%	ICU	[36]
Wang L	30-March	Wuhan	China	Retrospective case study	339	8	69 (7.4)	51%	Mortality	[53]
Guo T	27-March	Wuhan	China	Retrospective case study	187	8	58.5 (14.7)	51.3%	NA	[33]
Zheng C	27-March	Wuhan	China	Retrospective case study	55	7	57.2 (65.3)	43.6%	Severity	[66]
Chen T	26-March	Wuhan	China	Retrospective case study	274	9	58.7 (19.2)	37.6%	Mortality	[9]
Tang X	26-March	Wuhan	China	Retrospective case study	73	6	65.3 (11.1)	38.4%	NA	[49]
Shi S	25-March	Wuhan	China	Retrospective case study	416	9	60 (54.8)	50.7%	NA	[48]
TO K	23-March	Hong Kong	China	Observational cohort study	23	9	57.7 (27.5)	43.5%	Severity	[50]
Zhou Z	24-March	Chongqing	China	Retrospective case study	62	9	47.2 (13.4)	45.2%	Severity	[69]
Chen Z	24-March	Zhejiang	China	Retrospective case study	98	6	43 (17.2)	53.1%	NA	[27]
Wan S	21-March	Chongqing	China	Retrospective case study	135	9	46 (14.1)	46.7%	Severity	[51]
Cheng Y	20-March	Wuhan	China	Prospective cohort study	701	9	61.3 (15.5)	47.6%	NA	[28]
Luo S	20-March	Wuhan	China	Retrospective case study	183	5	53.8 (NA)	44%	NA	[42]
Deng Y	20-March	Wuhan	China	Retrospective case study	225	8	55.4 (11.5)	44.9%	Mortality	[30]
Arentz M	19-March	Washington	USA	Retrospective case study	21	5	68.3 (36.3)	48%	NA	[21]
Chen J	19-March	Shanghai	China	Retrospective case study	249	5	50.3 (20.7)	49.4%	NA	[25]
Cai Q	18-March	Shenzhen	China	Retrospective case study	80	9	47.9 (18.7)	56.2%	NA	[22]
Gao Y	17-March	Anhui	China	Retrospective case study	43	9	43.7 (11.8)	39.5%	Severity	[32]
Qian G	17-March	Zhejiang	China	Retrospective case study	91	5	47.8 (15.2)	59.3%	Severity	[45]
Mo P	16-March	Wuhan	China	Retrospective case study	155	8	54 (17.8)	44.5%	NA	[43]
Wang Z	16-March	Wuhan	China	Retrospective case study	69	7	46.3 (20)	54%	NA	[54]
Lo I	15-March	Macau	China	Retrospective case study	10	8	48.3 (27.4)	70%	Severity	[41]
Cheng Z	14-March	Shanghai	China	Retrospective case study	11	5	50.4 (15.5)	27.3%	NA	[29]
Hsih W	13-March	Taichung	Taiwan	Retrospective case study	2	5	45 (8.9)	50%	NA	[35]
Wu C	13-March	Wuhan	China	Retrospective case study	201	8	51.3 (12.6)	36.3%	Mortality	[55]
Qin C	12-March	Wuhan	China	Retrospective case study	452	9	57.3 (14.8)	48%	Severity	[46]
Zhao D	12-March	Wuhan	China	Case-control study	19	7	43.7 (21.5)	42.1%	NA	[65]
Liu K	11-March	Hainan	China	Retrospective case study	18	7	67.6 (3.3)	33.3%	NA	[38]
Zhou F	09-March	Wuhan	China	Retrospective case study	191	9	56.3 (15.5)	38%	Mortality	[67]
Xiong Y	07-March	Wuhan	China	Retrospective case study	42	5	49.5 (14.1)	40%	NA	[58]
Fan B	04-March	Singapore	Singapore	Retrospective case study	67	9	43.7 (14.1)	44.8%	ICU	[31]
Young B	03-March	Sengkang	Singapore	Descriptive case series	18	7	50.3 (31.1)	50%	NA	[62]
Wu J	29-February	Jiangsu	China	Retrospective case study	80	7	46.1 (15.4)	51.2%	NA	[56]
Li K	29-February	Chongqing	China	Retrospective case study	83	9	45.5 (12.3)	47%	Severity	[37]
Liu W	28-February	Wuhan	China	Retrospective case study	78	9	42.7 (17.8)	50%	NA	[39]
Yang W	26-February	Zhejiang	China	Retrospective case study	149	6	45.1 (13.3)	45.6%	NA	[60]
Wu J	25-February	Chongqing	China	Retrospective case study	80	6	44 (11)	48%	NA	[57]
Shi H	24-February	Wuhan	China	Retrospective case study	81	7	49.5 (11)	48%	NA	[47]
Yang X	24-February	Wuhan	China	Retrospective case study	52	9	59.7 (13.3)	33%	Mortality	[61]
Zhang J	23-February	Wuhan	China	Retrospective case study	138	9	56.3 (45.9)	49.3%	Severity	[64]
Zhou W	21-February	Wuhan	China	Retrospective case study	15	8	61.7 (9.6)	33.3%	Mortality	[68]
Xu X	19-February	Zhejiang	China	Retrospective case study	62	7	41.7 (14.8)	44%	NA	[59]
Pan F	13-February	Wuhan	China	Retrospective case study	21	6	40 (9)	74%	NA	[44]
Chang D	07-February	Beijing	China	Retrospective case study	13	6	38.7 (10.4)	23.1%	NA	[23]
Wang D	07-February	Wuhan	China	Retrospective case study	138	9	55.3 (19.2)	45.7%	ICU	[52]
Huang C	24-January	Wuhan	China	Prospective cohort study	41	9	49.3 (12.6)	27%	ICU	[1]

*All articles were published in 2020.

NA: not applicable.

### Pooled estimates of laboratory parameters: Single-arm meta-analysis

The final pooled estimates of single-arm meta-analysis included 52 eligible articles. The pooled mean of laboratory parameters and 95%CI among SARS-CoV-2 infected patients, including hematological, immunological, and inflammatory variables, is illustrated in Table 2. Our results depicted a wide variability between studies for each laboratory marker. Apart from immunoglobulins, IL-2R, and IL-8, significant heterogeneity was observed. Subgroup analysis by sample size and city of origin and sensitivity analysis failed to reveal the source of variation for each parameter. Additionally, meta-regression also rendered insignificant results.

**Table 2:** Pooled estimates of single-arm meta-analysis for laboratory parameters in COVID-19 patients.

Laboratory testing	Number studies	Sample size	Estimate	95% CI	P-value	Q	P-value	I^2^	T^2^
**CBC**									
White blood cells	47	5967	5.82	5.24, 6.40	<0.001	7136.1	<0.001	99.35	3.83
Neutrophil count	31	3814	3.70	3.48, 3.92	<0.001	525.8	<0.001	93.9	0.31
Lymphocyte count	45	6017	0.99	0.91, 1.08	<0.001	7645.2	<0.001	99.3	0.07
Monocyte count	18	2586	0.42	0.39, 0.44	<0.001	263.7	<0.001	93.5	0.003
Eosinophils count	4	546	0.02	0.01, 0.024	<0.001	10.6	0.014	71.6	0.0
Red blood cells	2	507	4.42	3.81, 4.67	<0.001	50.8	<0.001	98.03	0.095
Hemoglobin	26	3114	129.1	125.0, 133.3	<0.001	1504.3	<0.001	98.3	103.4
Platelet count	34	4347	178.4	171.9, 184.9	<0.001	390.2	<0.001	91.5	273.5
**Coagulation profile**									
Prothrombin time	22	3287	12.38	11.8, 12.9	<0.001	3415.7	<0.001	99.3	1.905
APTT	19	3023	31.8	30.2, 33.4	<0.001	1312.1	<0.001	98.6	11.96
Thrombin time	2	754	21.9	8.29, 35.57	0.002	1908.1	<0.001	99.94	96.86
D-dimer	27	3857	1.25	0.67, 1.82	<0.001	40947.5	<0.001	99.9	2.22
Fibrinogen	2	781	2.45	0.61, 4.29	0.009	46.19	<0.001	97.83	1.729
**Inflammatory markers**									
Ferritin	8	528	889.5	773.2, 1005.7	<0.001	16.61	0.020	57.8	14138.9
ESR	13	1013	37.85	29.07, 46.6	<0.001	692.4	<0.001	98.26	239.7
Procalcitonin	25	3010	0.10	0.07, 0.12	<0.001	3913.6	<0.001	99.3	0.003
C-reactive protein	36	4409	28.11	24.7, 31.4	<0.001	3432.1	<0.001	98.9	79.35
**Immunoglobulins**									
IgA	2	101	2.21	2.15, 2.27	<0.001	0.089	0.76	0.0	0.0
IgG	2	101	11.54	11.2, 11.8	<0.001	1.88	0.17	46.9	0.023
IgM	2	101	1.00	0.96, 1.04	<0.001	1.11	0.29	10.32	0.0
**Complement test**									
C3	2	101	0.95	0.80, 1.10	<0.001	28.02	<0.001	96.43	0.011
C4	2	101	0.24	0.21, 0.27	<0.001	28.08	<0.001	96.44	0.0
**Interleukins**									
IL-2R	2	101	762.3	732.4, 792.2	<0.001	0.33	0.56	0.0	0.0
IL-4	2	276	2.98	1.09, 4.87	0.002	958.765	<0.001	99.9	1.85
IL-6	12	926	11.56	9.82, 13.3	<0.001	144.7	<0.001	92.4	6.19
IL-8	2	101	18.4	17.08, 19.84	<0.001	1.54	0.21	35.3	0.39
IL-10	3	292	6.33	4.39, 8.27	<0.001	133.1	<0.001	98.4	2.89
TNF-α	3	292	6.72	1.33, 12.12	0.015	2933.6	<0.001	99.9	22.7
**Immune cells**									
CD4^+^ T cells	6	296	361.1	254.0, 468.2	<0.001	88.7	<0.001	94.3	15973.1
CD8^+^ T cells	5	285	219.6	157.1, 282.0	<0.001	46.17	<0.001	91.3	4437.2
T lymphocytes	2	167	704.3	254.5, 1154.0	0.002	27.6	<0.001	96.3	101500

Test of association: standardized mean difference, Random model. 95% CI: 95% confidence interval, Q statistic: a measure of weighted squared deviations that denotes the ratio of the observed variation to the within-study error, I^2^: the ratio of true heterogeneity to total observed variation, T^2^: Tau squared, and it is referred to the extent of variation among the effects observed in different studies. Laboratory markers (INR and B lymphocytes) were reported in only one study thus were not shown. CBC: Complete blood picture, APTT: Activated partial thromboplastin time, ESR: Erythrocyte sedimentation rate. Ig: immunoglobulin, IL-2R: Interleukin-2 receptor, TNF- α: tumor necrosis factor-alpha.

### Pooled estimates of laboratory parameters according to disease severity: Pairwise meta-analysis

Two-arms meta-analyses were then conducted for three pairwise comparisons; (1) Severe *versus* mild COVID, (2) ICU admitted patients *versus* the general ward, and (3) Expired *versus* survivors (Table 3).

**Table 3:** Pooled estimates of two-arms meta-analysis for laboratory parameters in COVID-19 patients.

Laboratory test	No of studies	Sample size		Effect size			Heterogeneity	
				SMD (95%CI)	OR (95% CI)	P-value	I^2^	P-value
**(A) Severity**		**Mild**	**Severe**					
White blood cells	**14**	1007	634	0.31 (0.11, 0.52)	**1.75 (1.21, 2.54)**	0.002	**62.9**	<0.001
Neutrophil count	**14**	959	599	0.53 (0.3, 0.76)	**2.62 (1.72, 3.97)**	<0.001	**67.61**	<0.001
Lymphocyte count	**16**	680	1128	-0.66 (-0.9, -0.41)	**0.30 (0.19, 0.47)**	<0.001	**77.36**	<0.001
Monocyte count	5	390	500	-0.08 (-0.23, 0.05)	0.86 (0.67, 1.12)	0.23	0.0	0.49
Hemoglobin	4	70	200	-0.22 (-0.51, 0.06)	0.67 (0.40, 1.12)	0.12	0.0	0.91
Platelet count	7	219	588	-0.32 (-0.47, -0.16)	**0.56 (0.42, 0.74)**	<0.001	0.0	0.76
Prothrombin time	6	215	521	0.33 (0.004, 0.67)	**1.82 (1.00, 3.33)**	0.047	**72.0**	0.003
APTT	5	146	386	-0.23 (-0.79, 0.33)	0.66 (0.24, 1.82)	0.42	85.5	<0.001
D-dimer	9	301	719	0.76 (0.53, 0.99)	**3.97 (2.62, 6.02)**	<0.001	**55.65**	0.021
Ferritin	2	297	176	1.003 (-0.08, 2.09)	6.17 (0.87, 43.9)	0.07	79.21	0.028
Fibrinogen	3	45	144	0.63 (0.27, 0.99)	**3.14 (1.64, 6.00)**	<0.001	0.0	0.81
ESR	2	302	277	0.26 (0.08, 0.44)	**1.60 (1.16, 2.22)**	0.004	0.0	0.43
Procalcitonin	**10**	565	716	0.86 (0.5, 1.22)	**4.76 (2.48, 9.14)**	<0.001	**86.1**	<0.001
C-reactive protein	**13**	605	928	1.02 (0.65, 1.4)	**6.36 (3.22, 12.5)**	<0.001	88.2	<0.001
IgA	2	355	301	0.13 (-0.03, 0.29)	1.27 (0.95, 1.69)	0.11	3.398	0.30
IgG	2	355	301	0.21 (-0.301, 0.72)	1.46 (0.58, 3.69)	0.41	88.3	0.003
IgM	2	355	301	-2.37 (-6.64, 1.89)	0.01 (0.00, 30.6)	0.27	99.56	<0.001
Complement 3	2	355	301	0.18 (-0.1, 0.47)	1.39 (0.83, 2.32)	0.20	64.70	0.09
Complement 4	2	355	301	0.13 (-0.16, 0.43)	1.27 (0.74, 2.16)	0.38	66.83	0.08
IL-4	2	355	301	1.01 (-0.85, 2.87)	6.25 (0.2, 181.1)	0.28	97.17	<0.001
IL-6	7	85	246	0.41 (0.014, 0.81)	**2.10 (1.02, 4.32)**	0.043	**84.38**	<0.001
IL-10	3	371	412	0.88 (0.43, 1.33)	**4.93 (2.18, 11.1)**	<0.001	**82.81**	0.003
TNF-α	3	371	412	0.6 (-0.17, 1.37)	2.97 (0.74, 11.9)	0.12	94.28	<0.001
CD4^+^ T cells	2	80	145	-1.87 (-2.39, -1.36)	**0.03 (0.01, 0.09)**	<0.001	29.8	0.23
CD8^+^ T cells	2	80	145	-1.8 (-2.12, -1.48)	**0.04 (0.02, 0.07)**	<0.001	0.0	0.71
**(B) Admission**		**Floor**	**ICU**					
White blood cells	3	64	149	0.85 (0.54, 1.15)	**4.67 (2.70, 8.10)**	<0.001	0.0	0.56
Neutrophil count	4	73	207	1.86 (0.59, 3.14)	**29.1 (2.9, 291.8)**	0.004	**93.14**	<0.001
Lymphocyte count	4	73	207	-0.81 (-1.36, -0.27)	**0.23 (0.09, 0.62)**	0.003	**68.59**	0.023
Monocyte count	3	60	179	-0.308 (-1.15, 0.53)	0.57 (0.13, 2.59)	0.47	83.77	0.002
Hemoglobin	2	22	86	-1.1 (-1.97, -0.24)	**0.14 (0.03, 0.64)**	0.012	**66.31**	0.08
Platelet count	4	73	207	-0.06 (-0.33, 0.2)	0.90 (0.56, 1.45)	0.64	0.0	0.54
Prothrombin time	3	64	149	0.43 (0.09, 0.76)	**2.18 (1.19, 3.99)**	0.012	14.28	0.31
APTT	3	64	149	-0.22 (-0.51, 0.07)	0.67 (0.40, 1.13)	0.14	0.0	0.78
D-dimer	3	64	149	0.79 (0.35, 1.24)	**4.19 (1.88, 9.35)**	<0.001	44.94	0.16
**(C) Mortality**		**Alive**	**Died**					
White blood cells	6	736	392	0.91 (0.61, 1.22)	**5.21 (3.00, 9.05)**	<0.001	**78.05**	<0.001
Neutrophil count	3	475	222	1.01 (0.4, 1.63)	**6.25 (2.05, 19.0)**	0.001	**90.9**	<0.001
Lymphocyte count	7	756	424	-0.85 (-1.28, -0.41)	**0.21 (0.10, 0.47)**	<0.001	**89.33**	<0.001
Monocyte count	4	483	229	-0.18 (-0.47, 0.1)	0.72 (0.43, 1.21)	0.21	57.48	0.070
Hemoglobin	5	600	271	0 (-0.15, 0.15)	1.00 (0.76, 1.31)	0.99	4.988	0.378
Platelet count	6	640	315	-0.46 (-0.71, -0.21)	**0.43 (0.28, 0.68)**	<0.001	**59.52**	0.030
Prothrombin time	6	640	315	0.64 (0.25, 1.03)	**3.19 (1.58, 6.47)**	0.001	83.0	<0.001
APTT	4	483	229	-0.096 (-0.51, 0.31)	0.83 (0.40, 1.75)	0.646	78.23	0.003
D-dimer	5	620	283	1.02 (0.85, 1.18)	**6.36 (4.72, 8.58)**	<0.001	10.63	0.34
Ferritin	3	338	211	0.94 (0.26, 1.62)	**5.50 (1.6, 18.83)**	0.006	**91.63**	<0.001
ESR	2	201	157	0.33 (0.08, 0.58)	**1.82 (1.16, 2.86)**	0.008	20.03	0.263
Procalcitonin	3	580	239	0.96 (0.43, 1.49)	**5.70 (2.18, 14.9)**	<0.001	**81.48**	0.005
C-reactive protein	4	591	331	1.08 (0.65, 1.52)	**7.09 (3.23, 15.5)**	<0.001	**87.31**	<0.001
IL-6	4	612	276	1.45 (1.11, 1.78)	**13.87 (7.6, 25.4)**	<0.001	**75.44**	0.007
CD4^+^ T cells	2	314	109	-0.67 (-1.01, -0.33)	**0.30 (0.16, 0.55)**	<0.001	44.57	0.17
CD8^+^ T cells	2	314	109	-0.832 (-1.1, -0.59)	**0.22 (0.15, 0.34)**	<0.001	0.0	0.423

Continuous Random-Effects model, SMD: Standardized mean difference, OR 95% CI: Odds ratio 95% confidence interval, I^2^: the ratio of true heterogeneity to total observed variation. APTT: Activated partial thromboplastin time, ESR: Erythrocyte sedimentation rate. Ig: immunoglobulin, IL: Interleukin, TNF-α: tumor necrosis factor-alpha.

Laboratory parameters of 16 eligible records were utilized to compare between severe and non-severe patients. Severe cohorts were more likely to have high blood levels of white blood cells (OR = 1.75, 95%CI = 1.21–2.54, *p* = 0.002), neutrophil count (OR = 2.62, 95%CI = 1.72–3.97, *p* <0.001), prothrombin time (OR = 1.82, 95%CI = 1.00–3.33, *p* = 0.047), D-dimer (OR = 3.97, 95%CI = 2.62–6.02, *p* <0.001), fibrinogen (OR = 3.14, 95%CI = 1.64–6.00, *p* <0.001), erythrocyte sedimentation rate (OR = 1.60, 95%CI = 1.16–2.22, *p* <0.001), procalcitonin (OR = 4.76, 95%CI = 2.48–9.14, *p* <0.001), IL-6 (OR = 2.10, 95%CI = 1.02–4.32, *p* = 0.043), and IL-10 (OR = 4.93, 95%CI = 2.18–11.1, *p* <0.001). In contrast, patients with normal lymphocyte count (OR = 0.30, 95%CI = 0.19–0.47, *p* <0.001), platelet count (OR = 0.56, 95%CI = 0.42–0.74, *p* <0.001), CD4^+^ T cells (OR = 0.04, 95%CI = 0.02–0.07, *p* <0.001), and CD8^+^ T cells (OR = 0.03, 95%CI = 0.01–0.09, *p* <0.001) were less likely to develop severe form of COVID-19 disease (Table 3A).

Significant heterogeneity was observed in eight of these parameters, namely WBC (I^2^ = 62.9%, *p* <0.001), neutrophil count (I^2^ = 67.6%, *p* <0.001), lymphocyte count (I^2^ = 77.4%, *p* <0.001), prothrombin time (I^2^ = 72%, *p* = 0.003), D-dimers (I^2^ = 55.6%, *p* = 0.021), procalcitonin (I^2^ = 86.1%, *p* <0.001), IL-6 (I^2^ = 84.4%, *p* <0.001), and IL-10 (I^2^ = 82.8%, *p* = 0.003).

### Pooled estimates of laboratory parameters according to ICU admission: Pairwise meta-analysis

A total of 4 eligible articles were recognized to include laboratory features of ICU and floor patients. Our data revealed having elevated levels of WBCs (OR = 5.21, 95%CI = 3.0–9.05, *p* <0.001), neutrophils (OR = 6.25, 95%CI = 2.05–19.0, *p* = 0.001), D-dimer (OR = 4.19, 95%CI = 1.88–9.35, *p* <0.001), and prolonged prothrombin time (OR = 2.18, 95%CI = 1.19–3.99, *p* = 0.012) were associated with increased odds of ICU admission, while normal lymphocyte count (OR = 0.23, 95%CI = 0.09–0.62, *p* = 0.003) and hemoglobin (OR = 0.14, 95%CI = 0.03–0.64, *p* = 0.012) conferred lower risk of ICU admission (Table 3B).

Remarkable heterogeneity was obvious in studies of neutrophil count (I^2^ = 93.1%, *p* <0.001), lymphocyte count (I^2^ = 68.5%, *p* = 0.023), and hemoglobin (I^2^ = 66.3%, *p* = 0.08). These parameters were enclosed in two to four studies; therefore, further tracing for the source of heterogeneity was not applicable.

### Pooled estimates of laboratory parameters according to mortality: Pairwise meta-analysis

Of the included articles, 7 studies contained separate results for laboratory testing in survival *versus* expired patients. As depicted in Table 3C, our data revealed increased odds of having elevated levels of WBC (OR = 5.21, 95%CI = 3.0–9.05, *p* <0.001), neutrophils (OR = 6.25, 95%CI = 2.05–19.0, *p* = 0.001), prothrombin time (OR = 3.19, 95%CI = 1.58–6.47, *p* = 0.001), D-dimer (OR = 6.36, 95%CI = 4.72–8.58, *p* <0.001), ferritin (OR = 5.50, 95%CI = 1.6–18.8, *p* = 0.006), ESR (OR = 1.82, 95%CI = 1.16–2.86, *p* = 0.008), procalcitonin (OR = 5.70, 95%CI = 2.18–14.9, *p* <0.001), CRP (OR = 7.09, 95%CI = 3.23–15.5, *p* <0.001), and IL-6 (OR = 13.87, 95%CI = 7.6–25.4, *p* <0.001) in expired cases. However, patients with normal lymphocyte count (0.21 (0.10, 0.47, p <0.001), platelet count (0.43 (0.28, 0.68, p <0.001), CD4^+^ T cells (OR = 0.30 (0.16, 0.55, p <0.001), and CD8^+^ T cells (OR = 0.22 (0.15, 0.34, p <0.001) had higher chance of survival (Table 3C).

Considerable heterogeneity was also noted in some of these parameters, namely WBC (I^2^ = 78.0%, *p* <0.001), neutrophilic count (I^2^ = 90.9%, *p* <0.001), lymphocyte count (I^2^ = 89.3%, *p* <0.001), platelet count (I^2^ = 59.5%, *p* = 0.030), ferritin (I^2^ = 91.6%, *p* <0.001), procalcitonin (I^2^ = 81.5%, *p* = 0.005), CRP (I^2^ = 87.3%, *p* <0.001), and IL-6 (I^2^ = 75.4%, *p* = 0.007). Given the small number of enrolled studies with discriminated data on patients who survived or died, we failed to identify the source of heterogeneity.

### Subgroup and sensitivity analysis

For the studies which included a comparison between mild and severe patients, subgroup and sensitivity analyses were performed for five laboratory markers (WBC, neutrophil count, lymphocyte count, procalcitonin, and CRP). First, to identify how each study affects the overall estimate of the rest of the studies, we performed leave-one-out sensitivity analyses. Results did not contribute to give explanations to heterogeneity. In contrast, subgroup analysis revealed homogeneity with certain categorizations. For WBCs lab results, heterogeneity was resolved on stratification by the origin of study population [Wuhan population: I^2^ = 73.4%, *p* = 0.002, other cities: I^2^ = 0%, *p* = 0.53] and month of publication [April: I^2^ = 74.5%, *p* = 0.001, February/March: I^2^ = 47.5%, *p* = 0.06]. Regarding neutrophilic count, the variance in the results resolved in articles with large sample size >50 patients (I^2^ = 46.2%, *p* = 0.06). Moreover, the degree of dissimilarities of procalcitonin results found in different studies was ameliorated in April publications (I^2^ = 41.5%, *p* = 0.16) and in those with low sample size (I^2^ = 0%, *p* = 0.80). Similarly, homogeneity was generated in CRP results in articles with low sample size (I^2^ = 0%, *p* = 0.58) (Table 4).

**Table 4:** Tracing the source of heterogeneity of laboratory markers in studies comparing mild and severe COVID-19 patients.

Lab test	Feature	Categories	Count of studies	Pooled estimates				Heterogeneity		Meta-regression			
				SMD	LL	UL	P-value	I^2^	P-value	Coefficient	LL	UL	P-value
**White blood cells**	Overall		14	0.317	0.113	0.52	0.002	62.90%	0.001				
	Origin of patients	Others	8	0.113	-0.083	0.308	0.26	0%	0.53	*Reference*			
		Wuhan	6	0.490	0.198	0.783	0.00	73.40%	0.002	0.31	0.03	0.58	0.029
	Sample size	≤50	5	0.164	-0.553	0.881	0.65	71.30%	0.007	*Reference*			
		>50	9	0.387	0.208	0.566	<0.001	52.60%	0.031	0.30	-0.10	0.72	0.14
	Publication month	Feb/Mar	8	0.251	0.039	0.464	0.021	47.50%	0.06	*Reference*			
		April	6	0.445	0.005	0.884	0.047	74.50%	0.001	0.11	-0.16	0.38	0.43
**Neutrophils**	Overall		14	0.534	0.306	0.762	<0.001	67.62%	<0.001				
	Origin of patients	Others	8	0.439	0.139	0.740	0.004	50.88%	0.047	*Reference*			
		Wuhan	6	0.632	0.280	0.985	<0.001	78.29%	<0.001	0.045	-0.21	0.30	0.20
	Sample size	≤50	5	0.286	-0.503	1.076	0.47	75.94%	0.002	*Reference*			
		>50	9	0.65	0.472	0.828	<0.001	46.2%	0.06	0.606	0.20	1.01	0.003
	Publication month	Feb/Mar	8	0.428	0.181	0.675	<0.001	54.4%	0.032	*Reference*			
		April	6	0.709	0.273	1.44	0.001	73.19%	0.002	0.312	0.06	0.55	0.014
**Lymphocytes**	Overall		16	-0.663	-0.909	-0.417	<0.001	77.36%	<0.001				
	Origin of patients	Others	9	-0.626	-0.962	-0.291	<0.001	66.51%	0.002	*Reference*			
		Wuhan	7	-0.710	1.097	-0.323	<0.001	85.72%	<0.001	0.092	-0.31	0.49	0.64
	Sample size	≤50	5	-0.506	-1.169	0.156	0.13	66.1%	0.019	*Reference*			
		>50	11	-0.714	-0.983	-0.444	<0.001	80.98%	<0.001	-0.342	-0.85	0.169	0.18
	Publication month	Feb/Mar	9	-0.452	-0.712	-0.192	<0.001	66.65%	0.002	*Reference*			
		April	7	-0.979	-1.354	-0.604	<0.001	70.53%	0.002	-0.572	-0.97	-0.17	0.006
**Procalcitonin**	Overall		10	0.868	0.508	1.228	<0.001	88.16%	<0.001				
	Origin of patients	Others	5	1.038	0.370	1.706	<0.001	86.16%	<0.001	*Reference*			
		Wuhan	5	0.686	0.331	1.041	<0.001	75.38%	0.003	-0.318	-0.97	0.33	0.34
	Sample size	≤50	3	0.768	0.334	1.203	<0.001	0%	0.80	*Reference*			
		>50	7	0.903	0.459	1.348	<0.001	88.62%	<0.001	0.054	-0.72	0.83	0.89
	Publication month	Feb/Mar	6	0.956	0.404	1.509	<0.001	91.51%	<0.001	*Reference*			
		April	4	0.757	0.409	1.105	<0.001	41.54%	0.16	-0.096	-0.80	0.61	0.78
**C-reactive protein**	Overall		13	1.027	0.65	1.40	<0.001	88.2%	<0.001				
	Origin of patients	Others	8	1.24	0.65	1.83	<0.001	87.8%	<0.001	*Reference*			
		Wuhan	5	0.389	0.30	1.07	<0.001	80.7%	<0.001	-0.58	-1.27	0.10	0.09
	Sample size	≤50	3	0.831	0.341	1.322	<0.001	0%	0.58	*Reference*			
		>50	10	1.08	0.651	1.512	<0.001	82.3%	<0.001	0.37	-0.55	1.29	0.42
	Publication month	Feb/Mar	8	1.014	0.502	1.525	<0.001	88.23%	<0.001	*Reference*			
		April	5	1.07	0.548	1.600	<0.001	75.1%	0.003	0.13	-0.59	0.86	0.71

SMD: Standardized mean difference, LL: lower limit, UL: upper limit, I^2^: the ratio of true heterogeneity to total observed variation. Significant values indicate significance at *P* < 0.05.

### Meta-regression analysis

Considering the number of the included studies with severity, ICU admission, and mortality data was rather small, we performed meta-regression analyses for only five parameters (mentioned above) in studies comparing mild and severe disease (Table 4).

For WBCs, higher difference between mild and severe cohorts was noted in Wuhan studies than other population (coefficient = 0.31, 95%CI = 0.03, 0.58, *p* = 0.029). Moreover, articles with larger sample size exhibited a wider variation of neutrophilic count between severe and non-severe cases (coefficient = 0.60, 95%CI = 0.20, 1.01, *p* = 0.003). For the same marker, later studies published in April also showed higher difference compared to those published in February and March (coefficient = 0.31, 95%CI = 0.06, 0.55, *p* = 0.014). In contrast, more reduction of lymphocytes was observed in April articles than earlier ones (coefficient = -0.57, 95%CI = -0.97, -0.17, *p* = 0.006).

### Publication bias

Publication bias was performed to the same five parameters with study count ≥10 (S1 Fig). Visual inspection of the funnel plots suggested symmetrical distribution for all laboratory parameters tested. The Egger test (*p* > 0.1) confirmed that there was no substantial evidence of publication bias; Egger’s regression *p* values were 0.44, 0.50, 0.68, 0.56, and 0.22 for WBC, neutrophil count, lymphocyte count, procalcitonin, and CRP, respectively.

### Decision tree and Receiver Operating Characteristic (ROC) curve

To identify predictors for severity, decision tree analysis was applied using multiple laboratory results. High performance of classification was found with the usage of a single parameter; neutrophilic count identified severe patients with 100% sensitivity and 81% specificity at a cut-off value of >3.74 identified by the specified decision tree model. Further analysis of the area under the curve of input data is shown in Table 5.

**Table 5:** Receiver operating characteristics results for severity of COVID-19.

Lab test	AUC	Threshold	Sensitivity	Specificity	P-value
WBC	0.801 ± 0.09	5.47	85.7	85.7	**0.007**
Neutrophil	0.831 ± 0.09	3.74	78.5	100	**0.003**
Lymphocyte	0.867 ± 0.06	0.98	81.2	87.5	**<0.001**
Platelets	0.836 ± 0.11	177.6	71.4	71.4	**0.035**
PT	0.583 ± 0.17	12.9	50.0	83.3	0.63
Procalcitonin	0.845 ± 0.09	0.06	80.0	90.0	**0.007**
D-dimer	0.876 ± 0.08	0.48	88.9	77.8	**0.007**
CRP	0.875 ± 0.08	38.2	84.6	92.3	**0.001**
IL-6	0.632 ± 1.6	22.9	71.4	71.4	0.40

AUC: area under the curve, WBC: white blood cells, PT: prothrombin time, CRP: C-reactive protein, IL-6: interleukin 6. Bold values indicate significance at *P* < 0.05.

### Trial sequential analysis

**Fig 2:**
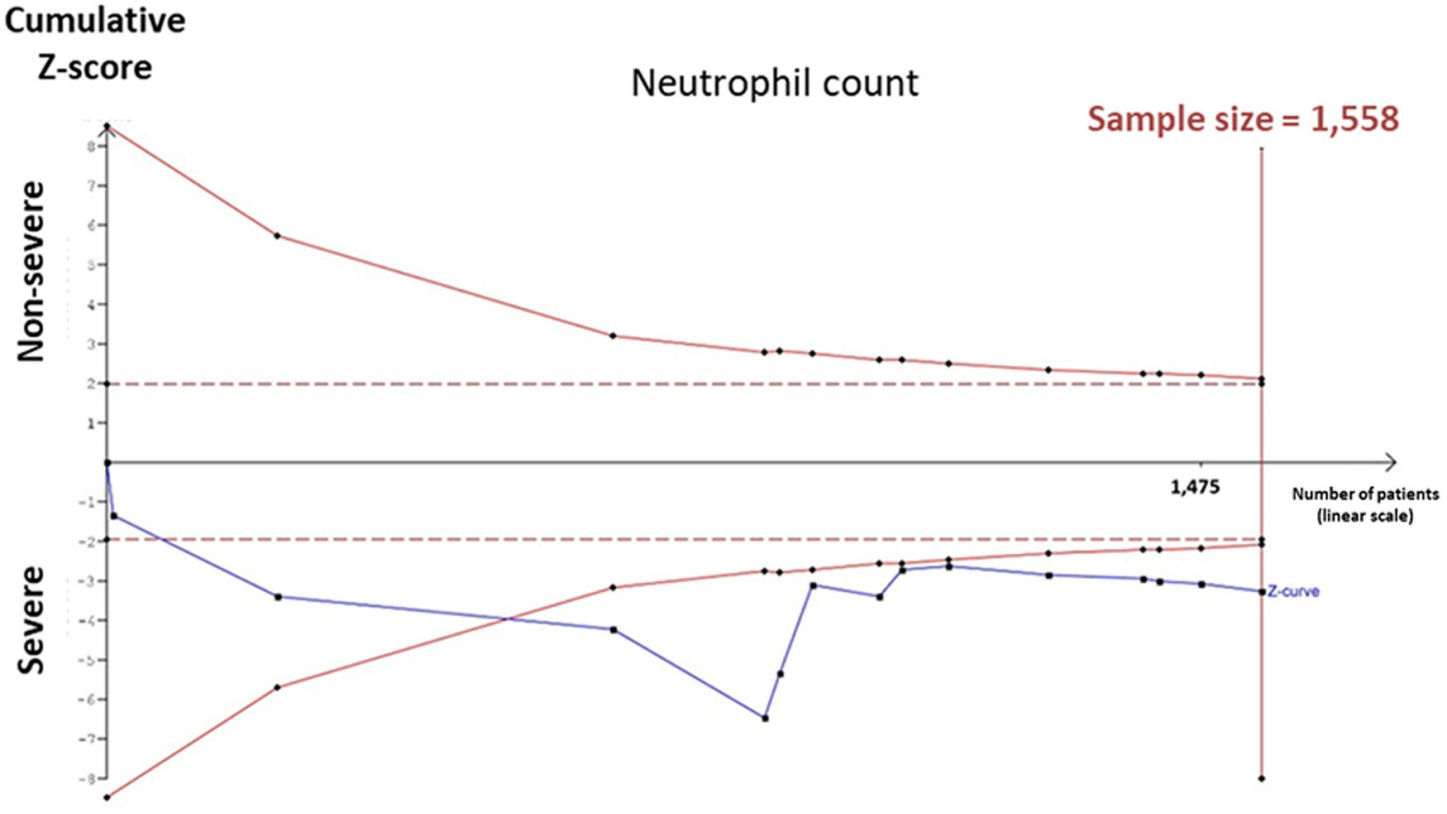
Trial sequential analysis. Trial sequential analysis (TSA) for the neutrophil count. The acquired sample size of the neutrophil count was 1558 subjects and the cumulative Z-curve crossed the monitoring boundary before reaching the required sample size (n = 540), suggesting that the cumulative proof was reliable, and no additional trials are required to achieve the significance.

As elaborated by the decision tree algorithm for the role of neutrophilic count on decision-making to discriminate between COVID-19 patients with a mild and severe presentation, TSA was employed on that particular laboratory parameter to test for the presence of sufficient studies from which results were drawn. The sample size of studies containing neutrophilic count information and classifying cohorts into mild and severe COVID-19 infection accounted for a total of 1,558 subjects. TSA illustrated crossing of the monitoring boundary by the cumulative Z-curve before reaching the required sample size, suggesting that the cumulative proof was acceptable, and no additional future studies are needed to authenticate the significances (Fig 2).

## Discussion

During the last few months, the prevalence of COVID-19 infection was increased daily among different countries overall in the world. Thus, the need to assess the disease severity and mortality are required to limit the pervasiveness of this pandemic [71]. A diverse of abnormal laboratory parameters including hematological, inflammatory as well as immunological markers thought to be raised throughout COVID-19 outbreak [2, 72]. In this comprehensive meta-analysis, our team attempted to interpret the distinct questions raised about the various spectrum of laboratory parameters associated with the severity and mortality of COVID-19. At the beginning of this workflow, our team investigated different hematological, inflammatory, and immunological variables of 6320 patients diagnosed with COVID-19. Our findings using random-effect models revealed increased levels of WBCs and neutrophil counts that were significantly associated with higher odds ratio among severe, ICU admission and Expired patients with COVID-19. On the contrary, the levels of lymphocyte and platelet counts were lowered among severe and expired patients with COVID-19. Also, we observed depletion in quantities of CD4^+^ T cells and CD8^+^ T cells among severe and mortality patients.

Nevertheless, in patients with the COVID-19 outbreak, the WBC count can vary [73]. Other reports indicated that leukopenia, leukocytosis, and lymphopenia have been reported, although lymphopenia appears most common [74, 75]. Another study supported that lymphopenia is an effective and reliable indicator of the severity and hospitalization in COVID-19 patients [76]. The additional report suggested that COVID-19 illness might be implicated with CD4^+^ and CD8^+^ T cells depletion through acting on lymphocytes, especially T lymphocytes [34]. A recent meta-analysis study discovered that the severity among COVID-19 patients might correlate with higher levels of WBCs count and lower levels of lymphocyte, CD4^+^ T cells, and CD8^+^ T cells counts [72]. In this respect, we could speculate that the depletion in the number of lymphocytes count is directly proportional with the severity of COVID-19 infection and the high survival rate of the disease is associated with the ability to renovate lymphocyte cells, particularly T lymphocytes which are crucial for destroying the infected viral particles [77]. During disease severity, remarkable thrombocytopenia was observed and confirmed by Lippi and his colleagues that revealed a reduction of platelet count among severe and died patients with COVID-19 supporting that thrombocytopenia could consider as an exacerbating indicator during the progression of the disease [78]. Therefore, our findings could support Shi et al. conclusion that high WBC count with lymphopenia could be considered as a differential diagnostic criterion for COVID-19 [79].

Considering coagulation profile, our team observed a prolonged in most coagulation markers among severe, ICU and expired patients, especially prothrombin time, fibrinogen, D-dimer, but with normal proportions of activated partial thromboplastin time (APTT) that could focus the light on the pathogenesis of COVID-19 infection through interfering with extrinsic coagulation pathway. A recently published report concluded similar findings in the form of observation of higher levels prothrombin time, D-dimer along fibrin degradation products among non-survival compared with survival patients [80].

Numerous studies illustrated the pathogenesis action of COVID-19 with the induction of cytokine storm throughout the progressive phase of the infection [72, 81, 82]. The generation of cytokine storm within COVID-19 patients required increased levels of IFN-γ and IL-1β that could stimulate the cellular response of T helper type 1 (Th1) which has a crucial function in the acceleration of specific immunity against COVID-19 outbreak [81]. Due to the elevated levels of IL-2R and IL-6 accompanied by the advancement of COVID-19, several cytokines secreted by T helper type 2 (Th2) cells that could neutralize the inflammatory responses including IL-4 and IL-10 [72, 81]. Our findings revealed a significantly associated with elevated levels of anti-inflammatory cytokines involving IL-6 and IL-10 among severe and expired patients with COVID-19. A recent study indicated a similar assumption with these findings and identified elevated levels of IL-6 and IL-10 among non-survived compared with survived patients [9]. Another confirmation of this conclusion is confirmed by a newly published meta-analysis report that indicated an exaggerated elevation of IL-6 and IL-10 throughout the severe level of COVID-19 infection [72].

Concerning the inflammatory markers associated with the COVID-19 pandemic, this comprehensive meta-analysis study observed higher concentrations of C-reactive protein (CRP) and procalcitonin besides elevated erythrocyte sedimentation rate (ESR) levels among severe and expired patients with COVID-19. Recently, Henry et al. established a meta-analysis survey and corroborated this finding with a higher significance of CRP and procalcitonin levels [72]. Other recent reports identified higher levels of CRP among severe patients with COVID-19 infection [76]. An additional meta-analysis survey established based on four recent articles indicated prolonged levels of procalcitonin among severe patients with COVID-19 [83]. In this respect, we might speculate the potential role of procalcitonin as a prognostic biomarker during the severe status of COVID-19. Finally, our team revealed increased levels of serum ferritin among non-survived patients compared with survived patients, and this significant outcome was observed in another meta-analysis study among severe and non-survival patients with COVID-19 infection [72].

This comprehensive meta-analysis confronted several limitations that raised throughout the processing of the outcomes. First, the insufficient laboratory data concerning the interest of design causing the increasing bias among different covariates. Second, the variation in the characteristics among different articles concerning the severity and survival of COVID-19. Third, the small sample sizes of some studies besides most of the concerned articles were established within China, especially Wuhan. Finally, there was an observed publication bias and heterogeneity in this comprehensive meta-analysis.

## Conclusion

In conclusion, several laboratory parameters could associate with the severity and mortality of COVID-19 infection and should be screened and measured continuously during the progression of this pandemic. These parameters included WBCs count, lymphocytes, platelet count, prothrombin time, D-dimer, and fibrinogen. Also, various interleukins could serve as anti-inflammatory markers such as IL-6, and IL-10 and should be evaluated. The estimation of other inflammatory biomarkers like CRP and procalcitonin could be helpful in the monitor the severity of the disease.

## Supporting information

S1 Table PRISMA checklist. (DOC)

S2 Table Reported timing of data collection and criteria of severity in eligible studies. (DOCX)

S1 Fig Publication bias. Funnel plot of standard error by the standardized difference in means for (A) White blood cells, (B) Neutrophil count, (C) Lymphocyte count, (D) Procalcitonin, and (E) C-reactive protein. The standard error provides a measure of the precision of the effect size as an estimate of the population parameter. It starts with zero at the top. Studies with smaller sample sizes produce less precise estimated effects with a broader base. The pooled estimated effects would be expected to scatter symmetrically around the total overall estimate of the meta-analysis (represented by the vertical line). Each circle represents a study (black circle). In the case of asymmetry, Duval and Tweedie’s trim and fill method predict the missing studies (red circle). Begg’s and Egger’s tests were performed. *P* values <0.1 were set to have a significant bias.
(TIF)
