# Supplementary material for: Diagnostic and prognostic value of hematological and immunological markers in COVID-19 infection: A meta-analysis of 6320 patients
Source: PLoS One. 2020 Aug 21;15(8):e0238160. doi: 10.1371/journal.pone.0238160 (PMC7446892; doi:10.1371/journal.pone.0238160)
Supplement: S2 Table — (DOCX) [file pone.0238160.s002.docx]

**S2 Table. Reported timing of data collection and criteria of severity in eligible studies.**

|  | **First author name** | **Time of collection of data** | **Criteria of severity** | **Ref.** |
| --- | --- | --- | --- | --- |
| **1** | Arentz, M. | Laboratory testing was reviewed at ICU  admission and on day 5. Chest radiographs were reviewed by an intensivist and a radiologist. Patient outcome data were  evaluated after 5 or more days of ICU care or at the time of death. | Severity was determined according to the Berlin Criteria. | [1] |
| **2** | Cai, Q. | From 30 January to 14 February 2020, laboratory-confirmed patients with COVID-19 were consecutively screened, and eligible patients were included in the FPV arm of the study. Patients who had initially been treated with antiviral therapy with LPV/RTV from 24 January to 30 January 2020 were screened, and eligible patients were included in the control arm of the study. | The inclusion criteria included: aged 16–75 years old; nasopharyngeal swabs samples tested positive for the novel coronavirus RNA; duration from disease onset to enrolment was less than 7 d; willing to take contraception during the study and within 7 d after treatment; and no difficulty in swallowing the pills.  The exclusion criteria included the following: severe clinical condition (meeting one of the following criteria: a resting respiratory rate greater than 30 per minute, oxygen saturation below 93%, oxygenation index (OI) < 300 mmHg (1 mmHg = 133.3 Pa), respiratory failure, shock, and/or combined failure of other organs that required ICU monitoring and treatment); chronic liver and kidney disease and reaching end stage; previous history of allergic reactions to FPV or LPV/RTV; pregnant or lactating women; women of a childbearing age with a positive pregnancy test, breastfeeding, miscarriage, or within 2 weeks after delivery; and participated in another clinical trial against SARSCoV-2 treatment currently or in the past 28 d. | [2] |
| **3** | Chang, L. | Not given | As exclusion criteria:  severe clinical condition is defined as (meeting one of the following criteria: a resting respiratory rate greater than 30 per minute, oxygen saturation below 93%, oxygenation index (OI) < 300 mmHg (1 mmHg= 133.3 Pa). | [3] |
| **4** | Chen, G. | On admission to hospital | According to the guidelines for diagnosis and management of COVID-19 (6^th^ edition, in Chinese) issued by the National Health Commission of China (9), 11 (52.4%) patients with percutaneous oxygen saturation (SpO2) of 93% or lower or respiratory rates of 30/min or greater on room air who required high-flow nasal cannula or non- invasive mechanical ventilation using the bilevel positive airway  pressure (BiPAP) mode to correct hypoxemia, were classified as having severe COVID-19, whereas 10 (47.6%) patients not reaching the criteria for severe COVID-19 were considered moderate. | [4] |
| **5** | Chen, J. | On admission to hospital | Not given | [5] |
| **6** | Chen, T. | During the hospital admission | All patients were diagnosed as having covid-19 and classified as being moderately, severely, or critically ill according to the Guidance for Corona Virus Disease 2019 (6th edition) released by the National Health Commission of China. | [6] |
| **7** | Chen, X. | Immediately after admission | Not given | [7] |
| **8** | Chen, Z. | We collected clinical and laboratory data for analysis, derived from an electronic medical record system, concerning patients admitted to our hospital from January 20, 2020 to February 17, 2020, who had been confirmed as having COVID-19 infection using RT-PCR. | Not given | [8] |
| **9** | Cheng, Y. | On admission | Not given | [9] |
| **10** | Cheng, Z. |  | Patients were considered to have suspected COVID-19 and were included in the study if  they had two of the following clinical features—fever, manifestations of pneumonia on imaging, and a normal or reduced total leukocyte count or total.  Patients With lymphocyte count—plus an epidemiologic history that included travel or a history of residence in Hubei province or other areas where continuous transmission of local cases occurred within 14 days before onset of symptoms, a history of contact with patients who had fever or respiratory symptoms and were from Hubei province or other areas with continuous transmission of local cases within 14 days before onset of the disease, or clustering or epidemiologic association with the new coronavirus infection [6]. | [10] |
| **11** | Deng, Y. | Not given | Not given | [11] |
| **12** | Fan, B. E. | On admission | Not given | [12] |
| **13** | Gao, Y. | Mild patients used data from their first laboratory test on admission, while severe patients had their most  recent laboratory test before their clinical diagnosis. | Not given | [13] |
| **14** | Guo, T. | On admission | Acute respiratory distress syndrome was defined according to the Berlin Definition. | [14] |
| **15** | He, Ruyuan | The laboratory results were obtained within the early  stage of the disease. The cases with incomplete data were excluded. | All patients were divided into severe and non-severe groups according to "Pneumonia diagnosis and treatment program for novel coronavirus infection (trial version 5)" issued by National Health Commission of the People’s Republic of China  (http://www.nhc.gov.cn/). The classification criteria were summarized as Table S1. | [15] |
| **16** | Hsih, Wen-Hsin | On admission | Not given | [16] |
| **17** | Huang, C. | On admission | Not given | [17] |
| **18** | Lei, S. | Not given | The time of COVID-19 onset was defined as the date when the first sign or symptom was noticed. Acute respiratory distress syndrome (ARDS) was defined according to the Berlin definition. [16] Acute cardiac injury was identified if the cardiac biomarkers (eg, hypersensitive troponin I, Creatine kinase MB) were above the 99% upper reference limit or new abnormalities were shown in electrocardiography and echocardiography. [5] Acute kidney injury was defined according to the KDIGO clinical practice guidelines. [17] Patients were admitted and transferred to intensive care unit (ICU) based on the progression patients admitted to ICU, myocardial enzymes, inflammatory stress, and blood gas analysis were determined on the day of ICU admission of organ dysfunction or the need of mechanical ventilation. | [18] |
| **19** | Li, K. | Not given | The severe/critical patients met any of the following condition: (1) respiratory rate of 30 breaths per minute or greater; (2) finger of oxygen saturation of 93%or less in a resting state; (3) arterial oxygen  tension (PaO2)/inspiratory oxygen fraction (FiO2) of 300mmHg or less (1 mm Hg = 0.133 kPa); (4) respiratory failure occurred and mechanical ventilation required; (5) shock occurred; and (6) patients with other organ failure needed intensive care unit monitoring and treatment | [19] |
| **20** | Liu, K. | Not given | New diagnostic criteria for coronavirus pneumonia  Suspected cases are determined in two cases. The first is "have any one of the epidemiological history, and meet any two of the clinical manifestations (fever and/or respiratory symptoms; with the imaging characteristics of the pneumonia mentioned above; the total number of white blood cells in the early stage of disease is normal or decreased, and the lymphocyte count is reduced). The second is "without a clear epidemiological history and conforms to 3 of the clinical manifestations (fever and/or respiratory symptoms; with the aforementioned imaging characteristics of pneumo-  nia; the total number of white blood cells in the early stage of disease is normal or decreased, and the lymphocyte count is reduced). Confirmed cases need to have a positive result of pathogenic evidence (real-time fluorescent RT-PCR detection of new coronavirus nucleic acid positive; or viral gene sequencing, highly homologous to known new coronavirus). | [20] |
| **21** | Liu, W. | On admission and after 2 weeks of admission to hospital. | After two weeks of hospitalization, disease evaluation and clinical typing were performed on  all patients according to the "Diagnosis and Treatment Protocol for Novel Coronavirus  Infection-Induced Pneumonia version 4 (trial)".[5] All patients were divided into a progression group or an improvement/stabilization group based on clinical typing results. Specific criteria were as follows: (1) progression group: common-type changed to severe- or critical-type, or death; severe-type changed to critical-type or death; critical-type progressed to death. (2) improvement/stabilization group: common-, severe-, and critical-types remained unchanged; severe-type changed to common-type; critical-type changed to severe- or common-type. | [21] |
| **22** | LIU X | Routine laboratory test of the coagulation variables and blood indexes were carried out before, during, and after the treatment | The diagnosis of severe case was made if patients met any of the following criteria: (1) respiratory rate ≥ 30 breaths/min; (2) SpO_2_ ≤ 93% while breathing room air; (3) PaO_2_/FiO_2_ ≤ 300 mmHg. A critically ill case was diagnosed if any of the following criteria was met: (1) respiratory failure which requiring mechanical ventilation; (2) shock; (3) combined with another organ failure and need to be admitted to ICU. | [22] |
| **23** | LO IL | Not given | "Severe" was classified if one of the following was present: a) dyspnea with a respiratory rate of ≥ 30 per minute, b) saturation ≤ 93%, and c) PaO2 / FiO2 ≤ 300mmHg; "Critical" was classified if one of the following was present: a) respiratory failure requiring mechanical ventilation, b) shock, and c) co-existing multiple organ failure requiring close monitoring in the Intensive Care Unit (ICU). | [23] |
| **24** | LUO S | Not given | Not given | [24] |
| **25** | MO P | On admission | serious illness was defined if satisfying at least one of the following items: (i) breathing rate ≥30/min; (ii) pulse oximeter oxygen saturation (SpO2)≤93% at rest; (iii) ration of partial pressure of arterial oxygen (PaO2) to fraction of inspired oxygen (FiO2)≤300mmHg (1mmHg=0.133kPa). Critical illness was defined if satisfying at least one of the following items: (i)respiratory failure occurred and received mechanical ventilation; (ii) shock; (iii) combined with failure of other organs and received care in the intensive care unit (ICU) | [25] |
| **26** | Pan F | Not given | Not given | [26] |
| **27** | Qian Q | Doctors who treated the patients collected and recorded the epidemiological characteristics by interviewing each patient on their activity history during the two weeks before symptoms onset or admission into hospital. | Patients were divided into the diagnosed as severe group and mild group according to national treatment guideline. | [27] |
| **28** | Qin C | Not given | he severity of COVID-19 was judged according to the Fifth Revised Trial Version of the Novel Coronavirus Pneumonia Diagnosis and Treatment Guidance.[9]Those who met the criterion as follows were defined as severe-type: 1. Respiratory distress with the respiratory rate over 30 per minute; 2. Oxygen saturation ≤ 93% in the resting state; 3. Arterial blood oxygen partial pressure (PaO2) / oxygen concentration (FiO2) ≤300mmHg | [28] |
| **29** | SHI H | Not given | Not given | [29] |
| **30** | Shi S | Cardiac biomarkers measured on admission were collected, including hs-TNI, CK-MB, and myohemoglobin. | Not given | [30] |
| **31** | Tang X | Not given | Not given | [31] |
| **32** | To KK | Not given | We defined severe disease as the need for supplemental oxygen, admission to the intensive care unit (ICU), or death | [32] |
| **33** | Wan S | Not given | The severe group had respiratory distress, RR≥30 beats/minute in a resting state, a mean oxygen saturation of ≤93%, and an arterial blood oxygen partial pressure (PaO2)/ oxygen concentration (FiO2) ≤300 mm Hg. The critical group had respiratory failure and required mechanical ventilation, the occurrence of shock, and the combined failure of other organs that required ICU monitoring and treatment. | [33] |
| **34** | Wang D | The date of disease onset was defined as the day when the symptom was noticed. Symptoms, signs, laboratory values, chest CT scan, and treatment measures during the hospital stay were collected. | Not given | [34] |
| **35** | Wang L | Not given | severe (dyspnea, respiratory frequency ≥30/minute, blood oxygen saturation ≤93%, PaO2/FiO2 ratio <300, and/or lung infiltrates >50% of the lung field within 24–48 h) and critical (respiratory failure requiring mechanical ventilation, shock or other organ failure that requires intensive care). | [35] |
| **36** | Wang Z | Not given | Not given | [36] |
| **37** | Wu C | The majority of the clinical data used in this study was collected from the first day of hospital admission unless indicated otherwise. | Not given | [37] |
| **38** | Wu J | Not given | Not given | [38] |
| **39** | Wu J | All patients completed CT examination within 2 days after admission, which was 7 ± 4 days from the onset of the disease. | Not given | [39] |
| **40** | Xiong Y | All 42 patients underwent initial CT average 4.5 days (range, 1–11 days) after the onset of symptoms. According to the National Health and Health Commission, they also underwent follow-up CT scans for evaluating the progression of the disease after a short period of standardized treatment. The mean interval time from initial to follow-up examinations was 7 days (range, 3–13 days). | Not given | [40] |
| **41** | Xu, X. | Medical records | Unable to estimate either the case fatality rate or the predictors of fatality. Moreover, the time since illness onset in some of our patients might be shorter than the observation period of 10 days, which could result in biases of clinical observation characteristics. | [41] |
| **42** | Yang, W. | Electronic medical records | The worldwide accepted pneumonia severity scoring systems, including Pneumonia Severity Index (PSI) and the CURB-65, were used to assess pneumonia severity. | [42] |
| **43** | Yang, X. | Electronic medical records | Critically ill patients were defined as those admitted to the intensive care unit (ICU) who required mechanical ventilation or had a fraction of inspired oxygen (FiO2) of at least 60% or more. Identification of critically ill patients was achieved by reviewing and analyzing admission logs and histories from all available electronic medical records and patient care resources. | [43] |
| **44** | Young, B. | Data from electronic health records were summarized | No patients presented with a severe acute respiratory distress syndrome, and only 1 required immediate supplemental oxygen. | [44] |
| **45** | Zhang, G. | Medical records | The severity of COVID-19 was defined based on the international guidelines for community-acquired pneumonia. | [45] |
| **46** | Zhang, J. | Medical records | Severity of COVID-19 was defined according to the diagnostic and treatment guideline for SARS-CoV-2 issued by Chinese National Health Committee (version 3-5). Severe COVID-19 was designated when the patients had one of the following criteria: (a) respiratory distress with respiratory frequency ≥30/min; (b) pulse oximeter oxygen saturation ≤93% at rest; and (c) oxygenation index (artery partial pressure of oxygen/inspired oxygen fraction, PaO2/FiO2) ≤ 300 mm Hg. | [46] |
| **47** | Zhao, D. | Medical records. The admission data of these patients were from Jan 23 to Feb 5, 2020 | All COVID-19 patients in this study didn’t have severe complication like ARDS or multiple organ failure which was reported in Wuhan patients or SARS patients during the admission.  There was lack of severe infection, to compare findings with severe infection with mild infection. | [47] |
| **48** | Zheng, C. | Medical records | We divided these 55 confirmed patients into three types: mild COVID-19– fever and mild respiratory symptoms, with pulmonary imaging such as computed tomography (CT) showing no obvious or only mild pneumonia; moderate COVID-19 – obvious respiratory symptoms such as fever and cough, and pulmonary CT indicates typical coronavirus pneumonia with overall lesions less than 30%; however, moderate patients have stable vital signs, and oxygen saturation > 93% without oxygen support; and severe COVID-19 –at least 1 of clinical features which including respiratory rate more than 30 breaths/minute at rest, oxygen saturation ≤ 93% without oxygen support, arterial oxygen partial pressure/fractional inspired oxygen (PaO2/FiO2) ≤ 300 mmHg, the total lesions on chest CT ≥ 30% or rapid progress ≥ 50% in next 72 hours (which were evaluated by two physicians and two radiologist). | [48] |
| **49** | Zhou, F. | Frequency of examinations was determined by the treating physician | The illness severity of COVID-19 was defined according to the Chinese management guideline for COVID-19 (version 6.0). | [49] |
| **50** | Zhou, W. | Retrospective | According to the Chinese government’s daily report.  Severe patients with advanced ARDS who could alleviate the pulmonary fibrosis. | [50] |
| **51** | Zhou, Z. | Retrospective | We defined patients with an onset of symptoms within 4 days as the early-stage group (18 men, 16 women; age range 20– 72 years old) and patients with an onset of symptoms within 5–7 days as the progressive-stage group (16 men, 12 women; age range 22–91 years old). | [51] |
| **52** | Zhu, Z. | Retrospective | All patients were diagnosed according to the guidelines for diagnosis and treatment for COVID-19 (Trail Version 6), and classified into mild, moderate, severe and critical type based on the severity of symptoms. Severe patients should meet at least one of the following criterions: First, shortness of breath with respiration rate (RR) ≥ 30 times/min. Second, oxygen saturation ≤ 93% in resting state. Third, partial pressure of arterial oxygen (PaO2)-to-fraction of inspired oxygen (FiO2) ratio ≤ 300 mm Hg. Obvious lesion progression >50% within 24-48 hours on pulmonary imaging were also recognized as severe cases. Critical cases were defined when one of the following conditions met: First, respiratory failure and require mechanical ventilation. Second, shock occurred. Third, combined with other organ failure and treated in intensive care unit. Mild and moderate cases were defined as non-severe group, while severe and critical patients were categorized as severe group in this study. | [52] |

**References**

1. Arentz, M., et al., Characteristics and Outcomes of 21 Critically Ill Patients With COVID-19 in Washington State. JAMA, 2020.

2. Cai, Q., et al., Experimental Treatment with Favipiravir for COVID-19: An Open-Label Control Study. Engineering, 2020.

3. Chang, et al., Epidemiologic and Clinical Characteristics of Novel Coronavirus Infections Involving 13 Patients Outside Wuhan, China. JAMA, 2020. **323**(11): p. 1092-1093.

4. Chen, G., et al., Clinical and immunological features of severe and moderate coronavirus disease 2019. J Clin Invest, 2020.

5. Chen, J., et al., Clinical progression of patients with COVID-19 in Shanghai, China. J Infect, 2020. **80**(5): p. e1-e6.

6. Chen, T., et al., Clinical characteristics of 113 deceased patients with coronavirus disease 2019: retrospective study. BMJ, 2020. **368**: p. m1091.

7. Chen, X., et al., Detectable serum SARS-CoV-2 viral load (RNAaemia) is closely correlated with drastically elevated interleukin 6 (IL-6) level in critically ill COVID-19 patients. Clin Infect Dis, 2020.

8. Chen, Z., et al., High-resolution computed tomography manifestations of COVID-19 infections in patients of different ages. Eur J Radiol, 2020. **126**: p. 108972.

9. Cheng, Y., et al., Kidney disease is associated with in-hospital death of patients with COVID-19. Kidney Int, 2020.

10. Cheng, Z., et al., Clinical Features and Chest CT Manifestations of Coronavirus Disease 2019 (COVID-19) in a Single-Center Study in Shanghai, China. AJR Am J Roentgenol, 2020: p. 1-6.

11. Deng, Y., et al., Clinical characteristics of fatal and recovered cases of coronavirus disease 2019 (COVID-19) in Wuhan, China: a retrospective study. Chin Med J (Engl), 2020.

12. Fan, B.E., et al., Hematologic parameters in patients with COVID-19 infection. Am J Hematol, 2020.

13. Gao, Y., et al., Diagnostic utility of clinical laboratory data determinations for patients with the severe COVID-19. J Med Virol, 2020.

14. Guo, T., et al., Cardiovascular Implications of Fatal Outcomes of Patients With Coronavirus Disease 2019 (COVID-19). JAMA Cardiol, 2020.

15. He, R., et al., The clinical course and its correlated immune status in COVID-19 pneumonia. Journal of Clinical Virology, 2020: p. 104361.

16. Hsih, W.-H., et al., Featuring COVID-19 cases via screening symptomatic patients with epidemiologic link during flu season in a medical center of central Taiwan. Journal of Microbiology, Immunology and Infection, 2020.

17. Huang, C., et al., Clinical features of patients infected with 2019 novel coronavirus in Wuhan, China. Lancet, 2020. **395**(10223): p. 497-506.

18. Lei, S., et al., Clinical characteristics and outcomes of patients undergoing surgeries during the incubation period of COVID-19 infection. EClinicalMedicine, 2020: p. 100331.

19. Li, K., et al., The Clinical and Chest CT Features Associated with Severe and Critical COVID-19 Pneumonia. Invest Radiol, 2020.

20. Liu, K., et al., Clinical features of COVID-19 in elderly patients: A comparison with young and middle-aged patients. J Infect, 2020.

21. Liu, W., et al., Analysis of factors associated with disease outcomes in hospitalized patients with 2019 novel coronavirus disease. Chin Med J (Engl), 2020.

22. Liu, X., et al., Potential therapeutic effects of dipyridamole in the severely ill patients with COVID-19. Acta Pharm Sin B, 2020.

23. Lo, I.L., et al., Evaluation of SARS-CoV-2 RNA shedding in clinical specimens and clinical characteristics of 10 patients with COVID-19 in Macau. Int J Biol Sci, 2020. **16**(10): p. 1698-1707.

24. Luo, S., X. Zhang, and H. Xu, Don't Overlook Digestive Symptoms in Patients With 2019 Novel Coronavirus Disease (COVID-19). Clin Gastroenterol Hepatol, 2020.

25. Mo, P., et al., Clinical characteristics of refractory COVID-19 pneumonia in Wuhan, China. Clin Infect Dis, 2020.

26. Pan, F., et al., Time Course of Lung Changes On Chest CT During Recovery From 2019 Novel Coronavirus (COVID-19) Pneumonia. Radiology, 2020: p. 200370.

27. Qian, G.Q., et al., Epidemiologic and Clinical Characteristics of 91 Hospitalized Patients with COVID-19 in Zhejiang, China: A retrospective, multi-centre case series. QJM, 2020.

28. Qin, C., et al., Dysregulation of immune response in patients with COVID-19 in Wuhan, China. Clin Infect Dis, 2020.

29. Shi, H., et al., Radiological findings from 81 patients with COVID-19 pneumonia in Wuhan, China: a descriptive study. Lancet Infect Dis, 2020.

30. Shi, S., et al., Association of Cardiac Injury With Mortality in Hospitalized Patients With COVID-19 in Wuhan, China. JAMA Cardiol, 2020.

31. Tang, X., et al., Comparison of Hospitalized Patients With ARDS Caused by COVID-19 and H1N1. Chest, 2020.

32. To, K.K., et al., Temporal profiles of viral load in posterior oropharyngeal saliva samples and serum antibody responses during infection by SARS-CoV-2: an observational cohort study. Lancet Infect Dis, 2020.

33. Wan, S., et al., Clinical features and treatment of COVID-19 patients in northeast Chongqing. J Med Virol, 2020.

34. Wang, D., et al., Clinical Characteristics of 138 Hospitalized Patients With 2019 Novel Coronavirus–Infected Pneumonia in Wuhan, China. JAMA, 2020. **323**(11): p. 1061-1069.

35. Wang, L., et al., Coronavirus disease 2019 in elderly patients: Characteristics and prognostic factors based on 4-week follow-up. J Infect, 2020.

36. Wang, Z., et al., Clinical Features of 69 Cases with Coronavirus Disease 2019 in Wuhan, China. Clin Infect Dis, 2020.

37. Wu, C., et al., Risk Factors Associated With Acute Respiratory Distress Syndrome and Death in Patients With Coronavirus Disease 2019 Pneumonia in Wuhan, China. JAMA Intern Med, 2020.

38. Wu, J., et al., Clinical Characteristics of Imported Cases of COVID-19 in Jiangsu Province: A Multicenter Descriptive Study. Clin Infect Dis, 2020.

39. Wu, J., et al., Chest CT Findings in Patients With Coronavirus Disease 2019 and Its Relationship With Clinical Features. Invest Radiol, 2020. **55**(5): p. 257-261.

40. Xiong, Y., et al., Clinical and High-Resolution CT Features of the COVID-19 Infection: Comparison of the Initial and Follow-up Changes. Invest Radiol, 2020.

41. Xu, X.W., et al., Clinical findings in a group of patients infected with the 2019 novel coronavirus (SARS-Cov-2) outside of Wuhan, China: retrospective case series. BMJ, 2020. **368**: p. m606.

42. Yang, W., et al., Clinical characteristics and imaging manifestations of the 2019 novel coronavirus disease (COVID-19):A multi-center study in Wenzhou city, Zhejiang, China. J Infect, 2020. **80**(4): p. 388-393.

43. Yang, X., et al., Clinical course and outcomes of critically ill patients with SARS-CoV-2 pneumonia in Wuhan, China: a single-centered, retrospective, observational study. Lancet Respir Med, 2020.

44. Young, B.E., et al., Epidemiologic Features and Clinical Course of Patients Infected With SARS-CoV-2 in Singapore. JAMA, 2020.

45. Zhang, G., et al., Clinical features and short-term outcomes of 221 patients with COVID-19 in Wuhan, China. J Clin Virol, 2020. **127**: p. 104364.

46. Zhang, J.J., et al., Clinical characteristics of 140 patients infected with SARS-CoV-2 in Wuhan, China. Allergy, 2020.

47. Zhao, D., et al., A comparative study on the clinical features of COVID-19 pneumonia to other pneumonias. Clin Infect Dis, 2020.

48. Zheng, C., et al., Risk-adapted Treatment Strategy For COVID-19 Patients. Int J Infect Dis, 2020. **94**: p. 74-77.

49. Zhou, F., et al., Clinical course and risk factors for mortality of adult inpatients with COVID-19 in Wuhan, China: a retrospective cohort study. Lancet, 2020. **395**(10229): p. 1054-1062.

50. Zhou, W., et al., Potential benefits of precise corticosteroids therapy for severe 2019-nCoV pneumonia. Signal Transduction and Targeted Therapy, 2020. **5**(1).

51. Zhou, Z., et al., Coronavirus disease 2019: initial chest CT findings. Eur Radiol, 2020.

52. Zhu, Z., et al., Clinical value of immune-inflammatory parameters to assess the severity of coronavirus disease 2019. International Journal of Infectious Diseases, 2020.
