# Supplementary figures and images for: Diagnostic and prognostic value of hematological and immunological markers in COVID-19 infection: A meta-analysis of 6320 patients
Source: PLoS One. 2020 Aug 21;15(8):e0238160. doi: 10.1371/journal.pone.0238160 (PMC7446892; doi:10.1371/journal.pone.0238160)

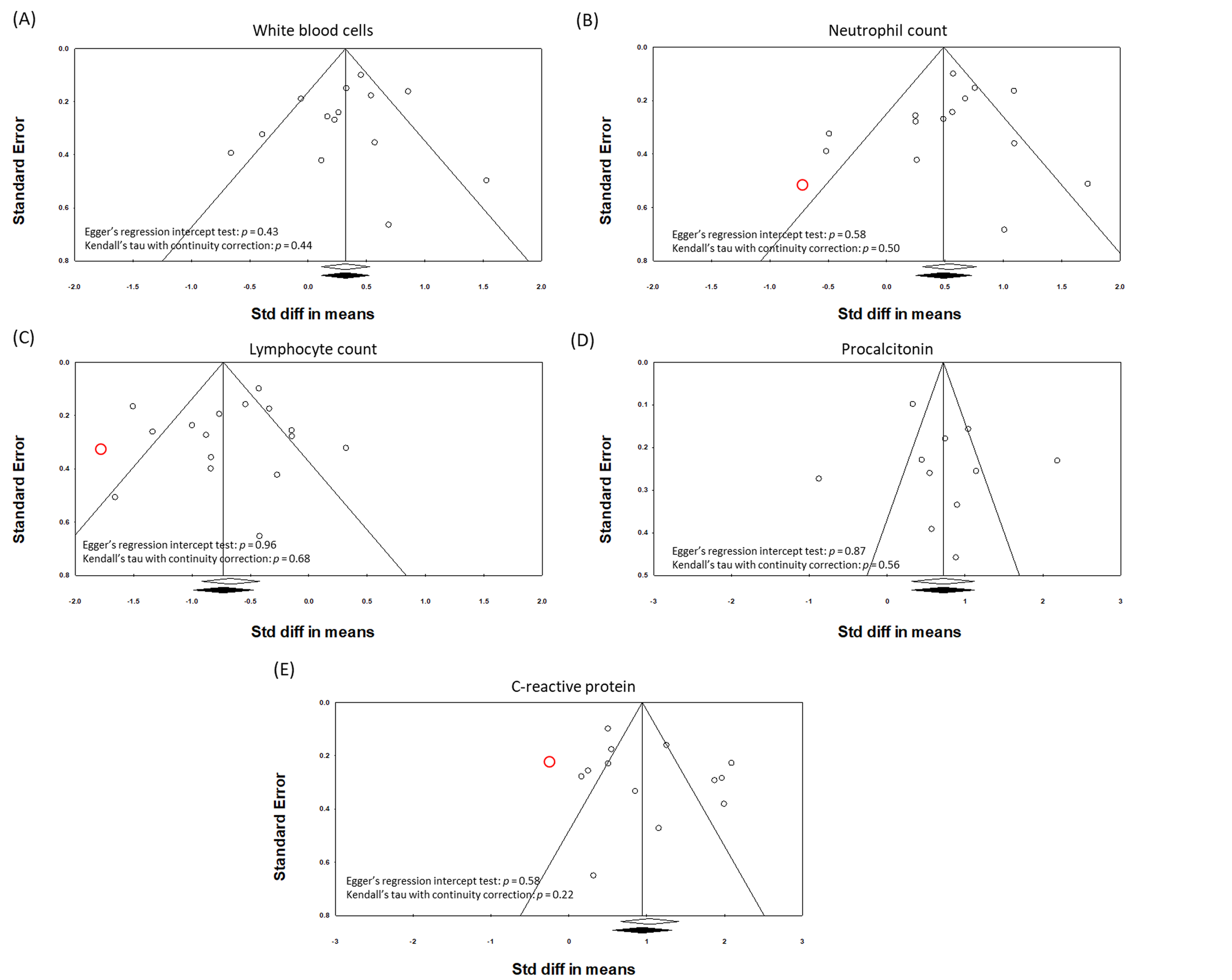

Supplement: S1 Fig — Funnel plot of standard error by the standardized difference in means for (A) White blood cells, (B) Neutrophil count, (C) Lymphocyte count, (D) Procalcitonin, and (E) C-reactive protein. The standard error provides a measure of the precision of the effect size as an estimate of the population parameter. It starts with zero at the top. Studies with smaller sample sizes produce less precise estimated effects with a broader base. The pooled estimated effects would be expected to scatter symmetrically around the total overall estimate of the meta-analysis (represented by the vertical line). Each circle represents a study (black circle). In the case of asymmetry, Duval and Tweedie’s trim and fill method predict the missing studies (red circle). Begg’s and Egger’s tests were performed. P values <0.1 were set to have a significant bias. (TIF) [file pone.0238160.s003.tif]
