# Supplementary material for: Diagnostic and prognostic value of hematological and immunological markers in COVID-19 infection: A meta-analysis of 6320 patients
Source: PLoS One. 2020 Aug 21;15(8):e0238160. doi: 10.1371/journal.pone.0238160 (PMC7446892; doi:10.1371/journal.pone.0238160)
Supplement: Attachment — Submitted filename: 2-Response sheet.docx [file pone.0238160.s004.docx]

**Response to reviewers' comments**

**(Manuscript ID PONE-D-20-13889R1)**

**“Diagnostic and prognostic value of hematological and immunological markers in COVID-19 infection: A meta-analysis of 6320 patients”**

**A point-by-point response to referee comments**

**Reviewer #2**

**None. Comments Addressed now.**

**Authors revised the suggestions and comments and proceed to improve and correct properly.**

**Author Response:**

**Thank you for your time.**

**Reviewer #3**

**The overall performance is good, still, many issues should be addressed**

**Author Response:**

**Thank you for your time and constructive comments. All suggested corrections have been addressed as follows:**

**1. Line 154:  Next, in the presence of individual patient data, single-armed observed values were converted to two-armed data to act as each other’s control group based on covariate information. Please provide Reference for articles presented with individual data**

**Author Response:**

**We followed reviewers’ suggestions and provided the references for articles presented with individual data (single-arm meta-analysis) in the results section (p.11; lines: 210-213).**

**2. Line 162: For severity pairwise comparison, estimates of SMD served as quantitative measures of the strength of evidence against the null hypothesis of no difference in the population between mild and severe COVID-19 manifestations.**

**Line 165: SMD of <0.2, 0.2-0.8, and >0.8 indicated mild, moderate, and severe strength.
Line 166:  For ICU admission, survival analysis, overall effect size estimates in SMD were then converted to the odds ratio (OR) with 95%CI for better interpretation by clinical domains.
What about moderate group defined by SMD 0.2-0.8?**

**Author Response:**

**Thank you for the remark. Earlier, we reported the data as SMD. The effect sizes of SMD were classified as small (<0.2), medium (0.2–0.8), or large (>0.8) magnitude. But for easier interpretation, we converted this value to OR. So, in the revised version, we deleted this statement to avoid confusion of the reader (lines 157-159).**

**3. Line 168: Decision tree to identify predictors for poor outcomes
In the manuscript, only severity was analyzed.**

**Author Response:**

**Thank you for the valued query. Decision tree analysis required large sample size (≥10 studies) (this issue added and highlighted to be clear for the readers: p.9; line 163), therefore, analysis was applied for WBC, neutrophil count, lymphocyte count, procalcitonin, and CRP variables which was reported in 10-16 studies comparing mild versus severe. There were not enough data available in the papers reporting ICU admission or mortality, thus we were not able to apply decision tree in these comparisons.**

**4. Line169:  Using laboratory features for clinical prediction, the decision tree algorithm was employed to identify the key risk factors attributed to severe COVID-19 infection.
For Risk factor? Not for cutoff value? No matter whatever it is, please provide the decision tree results as supplemental material.**

**Author Response:**

**Kindly, the decision tree was run by codes through entering first the data as follows:**

**Then through several process, the outcome yield as follows:**

**As shown it will be difficult to illustrate these outcomes as supplementary data as not all readers will digest it. Then subsequently, the accuracy of the model was measured by the Area Under the Receiver Operating Characteristic (ROC) Curve (AUC), which depicts the true positive rate versus the false positive rate at various discrimination thresholds which has been illustrated in Table 5.**

**5. Line 216: Ultimately, a total of 52 eligible articles were included for the quantitative synthesis of this meta-analysis study, with 52 records represented single-arm analysis, 16 records represented two-arms**

**Sixteen included articles have both single- and two-arms design?
And please specify the arms here, what is it?**

**Author Response:**

**Thank you for raising this point. To facilitate following the authors in their elaboration, the number of studies for each pairwise comparison was added in the text (lines 210-213) and articles are referenced as well. Kindly, according to available data,  in the case of a systematic review of a single outcome without comparison, it would be appropriate to report descriptive summary statistics in the form of mean, standard deviation, and range for parametric continuous measures….etc. If the demographics of the study populations and inclusion/exclusion are relatively similar between the studies, this may warrant a meta-analysis of weighted proportions, using a random-effects model to pool one-arm cohort studies. While in two-arm meta-analysis there are two cohorts (control and the group in which the intervention/outcome measured against) in each study included in the meta-analysis.**

**6. Line 236:  descriptive case series, and one case-control study.
Line 212 excluded for being case records (n = 44)
What is difference between case series and case records here?**

**Author Response:**

**Thank you for your query. The case records refer to case reports. We changed the term as suggested (p.11; line 205).**

**7. Table 1
1) Journal name and Publication date are not necessary to be included.
But, patients’ gender, age, outcomes should be provided.**

**Author Response:**

**The authors followed the referee suggestion and added the age, sex, and outcome comparison in table 1. Thank you for the valued suggestion. The authors just keep the date of publication (day/month) to give a general idea about the chronological order of the included studies and deleted the “year” column as all included studies were in 2020.**

**8. Table 2**

**What are the outcome measures here to predict by biological parameters?**

**Author Response:**

**Kindly, as mentioned above (reply to query 5), Table 2 is a single-arm meta-analysis without comparison of two groups, it describes the pooled estimates of laboratory findings (parameters) in COVID-19 patients in the included studies.**

**9.Table 5
Specify the outcome “****severity of COVID19” in the title.**

**Author Response:**

**Thank you for the remark. The outcome “severity of COVID19” has been specified accordingly.**

**10.Figure 2.
RSA sample size or RIS should be notified.**

**Author Response:**

**Thank you for the remark. It has been notified in the legend of Fig.1. (reaching the required sample size (n = 540) (added and highlighted p.27; line 391).**

**11.Subgroup analyses
why not conduct subgroup analyses by quality? diagnosis criteria for severity? or methods of biological parameters measurements?**

**Author Response:**

**Kindly, the detailed pooled estimates for laboratory parameters with effect estimates, and according to disease severity, ICU admission, and mortality were elaborated in detail (p.17-21), followed by tracing the source of heterogeneity of laboratory markers in studies comparing mild and severe COVID-19 patients (Table 4), and then the meta-regression analysis. Regarding the subgrouping by quality, this is a valued suggestion will be considered in future work as this will need exhausted work from the scratch to yield this specified entity. To the authors knowledge, they thought that the basic required analysis for the present meta-analysis that give almost comprehensive picture about the aim of the work has been almost covered and we really appreciate this valued suggestion.**

**12.Time point of collection of lab parameters and clinic symptoms?**

**Author Response:**

**Some papers reported the data at time of presentation in the Emergency department, others not specified. Some articles collected data from medical archives (retrospective). Regarding the criteria of assessment for severity, it was not supplied in all papers as well. Therefore, to illustrate what is reported in each paper, we added a supplementary table which includes these two elements: “Table S2. Reported timing of data collection and criteria of severity in eligible studies”.**

***Thank you for your time and efforts***
