# Supplementary material for: Diagnostic and prognostic value of hematological and immunological markers in COVID-19 infection: A meta-analysis of 6320 patients
Source: PLoS One. 2020 Aug 21;15(8):e0238160. doi: 10.1371/journal.pone.0238160 (PMC7446892; doi:10.1371/journal.pone.0238160)
Supplement: Peer review history

# Peer review: Decision Letter 0

**DOI:** 10.1371/journal.pone.0238160.r001

**Authors:** Farhat Afrin

12 Jun 2020

PONE-D-20-13889

Diagnostic and prognostic value of hematological and immunological markers in COVID-19 infection: A meta-analysis of 6320 patients

PLOS ONE

Dear Dr. Fawzy,

Thank you for submitting your manuscript to PLOS ONE. After careful consideration, we feel that it has merit but does not fully meet PLOS ONE’s publication criteria as it currently stands. Therefore, we invite you to submit a revised version of the manuscript that addresses the points raised during the review process.

The manuscript highlights the *L. infantum* loads and inflammation in the genital tract of naturally infected dogs in an endemic area of Brazil by qPCR and IHC. Besides vertical transmission, it also suggests venereal transmission from both the sexes as also suggested by other studies. The positive aspect is the higher number of animals used in the present study; otherwise, there are no new findings compared to similar studies conducted in the past. 

The manuscript cannot be accepted as it presently stands. There are serious concerns that need to be addressed as elaborated in Reviewer’s comments. 

Further, the authors should incorporate the following suggestions:

1. Every method should be supported by a reference.
2. Few references need to be updated. 
3. The authors have mentioned the results of other studies at several instances while discussing their findings, which is confusing. The exact values from other studies need not be mentioned every time.
4. The dogs were naturally infected and of different ages. The authors should discuss why they found similar parasite loads in the different genital organs (testis, epididymis, vulva and vagina) and significantly low in prostrate and uterus, unlike other studies. They have mentioned the anatomic proximity of the organs. 
5. The title should be modified to bring out the crux of the study; it is too detailed at the moment.

Please submit your revised manuscript by Jul 27 2020 11:59PM. If you will need more time than this to complete your revisions, please reply to this message or contact the journal office at plosone@plos.org. When you're ready to submit your revision, log on to https://www.editorialmanager.com/pone/ and select the 'Submissions Needing Revision' folder to locate your manuscript file.

Please include the following items when submitting your revised manuscript:

- A rebuttal letter that responds to each point raised by the academic editor and reviewer(s). You should upload this letter as a separate file labeled 'Response to Reviewers'.
- A marked-up copy of your manuscript that highlights changes made to the original version. You should upload this as a separate file labeled 'Revised Manuscript with Track Changes'.
- An unmarked version of your revised paper without tracked changes. You should upload this as a separate file labeled 'Manuscript'.

If you would like to make changes to your financial disclosure, please include your updated statement in your cover letter. Guidelines for resubmitting your figure files are available below the reviewer comments at the end of this letter.

If applicable, we recommend that you deposit your laboratory protocols in protocols.io to enhance the reproducibility of your results. Protocols.io assigns your protocol its own identifier (DOI) so that it can be cited independently in the future. For instructions see: http://journals.plos.org/plosone/s/submission-guidelines#loc-laboratory-protocols

We look forward to receiving your revised manuscript.

Kind regards,

Farhat Afrin, Ph.D.

Academic Editor

PLOS ONE

Additional Editor Comments:

The manuscript highlights the L. infantum loads and inflammation in the genital tract of naturally infected dogs in an endemic area of Brazil by qPCR and IHC. Besides vertical transmission, it also suggests venereal transmission from both the sexes as also suggested by other studies. The positive aspect is the higher number of animals used in the present study; otherwise, there are no new findings compared to similar studies conducted in the past.

The manuscript cannot be accepted as it presently stands. There are serious concerns that need to be addressed as elaborated in Reviewer’s comments.

Further, the authors should incorporate the following suggestions:

1) Every method should be supported by a reference.

2) Few references need to be updated.

3) The authors have mentioned the results of other studies at several instances while discussing their findings, which is confusing. The exact values from other studies need not be mentioned every time.

4) The dogs were naturally infected and of different ages. The authors should discuss why they found similar parasite loads in the different genital organs (testis, epididymis, vulva and vagina) and significantly low in prostrate and uterus, unlike other studies. They have mentioned the anatomic proximity of the organs.

5) The title should be modified to bring out the crux of the study; it is too detailed at the moment.

Journal Requirements:

When submitting your revision, we need you to address these additional requirements.

1. Please ensure that your manuscript meets PLOS ONE's style requirements, including those for file naming. The PLOS ONE style templates can be found at

https://journals.plos.org/plosone/s/file?id=wjVg/PLOSOne_formatting_sample_main_body.pdf and

https://journals.plos.org/plosone/s/file?id=ba62/PLOSOne_formatting_sample_title_authors_affiliations.pdf

2. We note that Figure 1B in your submission contain [map/satellite] images which may be copyrighted. All PLOS content is published under the Creative Commons Attribution License (CC BY 4.0), which means that the manuscript, images, and Supporting Information files will be freely available online, and any third party is permitted to access, download, copy, distribute, and use these materials in any way, even commercially, with proper attribution. For these reasons, we cannot publish previously copyrighted maps or satellite images created using proprietary data, such as Google software (Google Maps, Street View, and Earth). For more information, see our copyright guidelines: http://journals.plos.org/plosone/s/licenses-and-copyright.

We require you to either (1) present written permission from the copyright holder to publish these figures specifically under the CC BY 4.0 license, or (2) remove the figures from your submission:

2.1.    You may seek permission from the original copyright holder of Figure 1B to publish the content specifically under the CC BY 4.0 license.

We recommend that you contact the original copyright holder with the Content Permission Form (http://journals.plos.org/plosone/s/file?id=7c09/content-permission-form.pdf) and the following text:

“I request permission for the open-access journal PLOS ONE to publish XXX under the Creative Commons Attribution License (CCAL) CC BY 4.0 (http://creativecommons.org/licenses/by/4.0/). Please be aware that this license allows unrestricted use and distribution, even commercially, by third parties. Please reply and provide explicit written permission to publish XXX under a CC BY license and complete the attached form.”

Please upload the completed Content Permission Form or other proof of granted permissions as an "Other" file with your submission.

In the figure caption of the copyrighted figure, please include the following text: “Reprinted from [ref] under a CC BY license, with permission from [name of publisher], original copyright [original copyright year].”

2.2.    If you are unable to obtain permission from the original copyright holder to publish these figures under the CC BY 4.0 license or if the copyright holder’s requirements are incompatible with the CC BY 4.0 license, please either i) remove the figure or ii) supply a replacement figure that complies with the CC BY 4.0 license. Please check copyright information on all replacement figures and update the figure caption with source information. If applicable, please specify in the figure caption text when a figure is similar but not identical to the original image and is therefore for illustrative purposes only.

The following resources for replacing copyrighted map figures may be helpful:

USGS National Map Viewer (public domain): http://viewer.nationalmap.gov/viewer/

The Gateway to Astronaut Photography of Earth (public domain): http://eol.jsc.nasa.gov/sseop/clickmap/

Maps at the CIA (public domain): https://www.cia.gov/library/publications/the-world-factbook/index.html and https://www.cia.gov/library/publications/cia-maps-publications/index.html

NASA Earth Observatory (public domain): http://earthobservatory.nasa.gov/

Landsat: http://landsat.visibleearth.nasa.gov/

USGS EROS (Earth Resources Observatory and Science (EROS) Center) (public domain): http://eros.usgs.gov/#

Natural Earth (public domain): http://www.naturalearthdata.com/

[Note: HTML markup is below. Please do not edit.]

Reviewers' comments:

Reviewer's Responses to Questions

**Comments to the Author**

1. Is the manuscript technically sound, and do the data support the conclusions?

The manuscript must describe a technically sound piece of scientific research with data that supports the conclusions. Experiments must have been conducted rigorously, with appropriate controls, replication, and sample sizes. The conclusions must be drawn appropriately based on the data presented. 

Reviewer #1: Yes

**********

2. Has the statistical analysis been performed appropriately and rigorously? 

Reviewer #1: Yes

**********

3. Have the authors made all data underlying the findings in their manuscript fully available?

The PLOS Data policy requires authors to make all data underlying the findings described in their manuscript fully available without restriction, with rare exception (please refer to the Data Availability Statement in the manuscript PDF file). The data should be provided as part of the manuscript or its supporting information, or deposited to a public repository. For example, in addition to summary statistics, the data points behind means, medians and variance measures should be available. If there are restrictions on publicly sharing data—e.g. participant privacy or use of data from a third party—those must be specified.

Reviewer #1: Yes

**********

4. Is the manuscript presented in an intelligible fashion and written in standard English?

PLOS ONE does not copyedit accepted manuscripts, so the language in submitted articles must be clear, correct, and unambiguous. Any typographical or grammatical errors should be corrected at revision, so please note any specific errors here.

Reviewer #1: Yes

**********

5. Review Comments to the Author

Please use the space provided to explain your answers to the questions above. You may also include additional comments for the author, including concerns about dual publication, research ethics, or publication ethics. (Please upload your review as an attachment if it exceeds 20,000 characters)

Reviewer #1: The review is written well. But it needs several refinements.

1. The abstract (results): The values of parameters must be given with indicative conclusions. The reader will read the abstract before deciding to read the full article or not. Unfortunately the abstract is not well written.

2. Too many tables and same data is presented in the figures 2 and 3. I strongly feel that that Figure 2 and 3 are not represented correctly. Each panel of these figures deserve a separate independent figure. The journal also advises how to format a figure/graphs. The authors should use that format. The current form of these two important figures (having so many panels within) are not readable and have been made irrelevant.

3. The conclusions are also not crisp and clear. and need rephrasing with clear message. The words like various cytokines, markers, means nothing. We all know that all the cytokines and inflammatory markers are high in COVID-19, but this review can only be relevant if the author give clear message, eg. If D-Dimer is more significant or IL-6 value. (This is just an illustration)

**********

6. PLOS authors have the option to publish the peer review history of their article (what does this mean?). If published, this will include your full peer review and any attached files.

If you choose “no”, your identity will remain anonymous but your review may still be made public.

**Do you want your identity to be public for this peer review?** For information about this choice, including consent withdrawal, please see our Privacy Policy.

Reviewer #1: Yes: Prof. Sarman Singh

[NOTE: If reviewer comments were submitted as an attachment file, they will be attached to this email and accessible via the submission site. Please log into your account, locate the manuscript record, and check for the action link "View Attachments". If this link does not appear, there are no attachment files.]

While revising your submission, please upload your figure files to the Preflight Analysis and Conversion Engine (PACE) digital diagnostic tool, https://pacev2.apexcovantage.com/. PACE helps ensure that figures meet PLOS requirements. To use PACE, you must first register as a user. Registration is free. Then, login and navigate to the UPLOAD tab, where you will find detailed instructions on how to use the tool. If you encounter any issues or have any questions when using PACE, please email PLOS at figures@plos.org. Please note that Supporting Information files do not need this step.

# Author comment: Author response to Decision Letter 0

**DOI:** 10.1371/journal.pone.0238160.r002

14 Jun 2020

Journal Requirements

1. Please ensure that your manuscript meets PLOS ONE's style requirements, including those for file naming. 

Response

The authors ensure that our manuscript meets PLOS ONE's style requirements, including those for file naming. 

2. We note that Figure 1B in your submission contain [map/satellite] images which may be copyrighted.

Response:

The map in Fig 1 B is curated by the authors using Tableau 2020.1 Desktop Professional Edition (https://www.tableau.com/). These data were provisded in the figure legend to clarify this issue and cite the vendor in the text.

Reviewer #1 

The review is written well. But it needs several refinements.

Author Response: 

Dear Prof. Sarman Singh

We appreciate the time put in reviewing this manuscript. Thank you for the constructive comments. The authors followed it.

1. The abstract (results): The values of parameters must be given with indicative conclusions. The reader will read the abstract before deciding to read the full article or not. Unfortunately, the abstract is not well written.

Author Response: 

Thank you for the remark. The abstract has been revised to be attractive for future readers.

2. Too many tables and same data is presented in the figures 2 and 3. I strongly feel that that Figure 2 and 3 are not represented correctly. Each panel of these figures deserve a separate independent figure. The journal also advises how to format a figure/graphs. The authors should use that format. The current form of these two important figures (having so many panels within) are not readable and have been made irrelevant.

Author Response: 

We followed reviewers’ suggestions. We removed Figure 2 as the representative data included in the tables and let figure 3 as supplementary material (Figure S1) based on the referee's valued suggestion. The authors only represented the sequential trial analysis in a separate main figure (Figure 2).

3. The conclusions are also not crisp and clear. and need rephrasing with clear message. The words like various cytokines, markers, means nothing. We all know that all the cytokines and inflammatory markers are high in COVID-19, but this review can only be relevant if the author give clear message, e.g. If D-Dimer is more significant or IL-6 value. (This is just an illustration)

Author Response: 

Thank you for the remark. The conclusion has been revised and highlighted according to the valued suggestion.

# Peer review: Decision Letter 1

**DOI:** 10.1371/journal.pone.0238160.r003

**Authors:** Farhat Afrin

15 Jul 2020

PONE-D-20-13889R1

Diagnostic and prognostic value of hematological and immunological markers in COVID-19 infection: A meta-analysis of 6320 patients

PLOS ONE

Dear Dr. Fawzy,

Thank you for submitting your manuscript to PLOS ONE. After careful consideration, we feel that it has merit but does not fully meet PLOS ONE’s publication criteria as it currently stands. Therefore, we invite you to submit a revised version of the manuscript that addresses the points raised during the review process.

Please submit your revised manuscript by Aug 29 2020 11:59PM. If you will need more time than this to complete your revisions, please reply to this message or contact the journal office at plosone@plos.org. When you're ready to submit your revision, log on to https://www.editorialmanager.com/pone/ and select the 'Submissions Needing Revision' folder to locate your manuscript file.

Please include the following items when submitting your revised manuscript:

- A rebuttal letter that responds to each point raised by the academic editor and reviewer(s). You should upload this letter as a separate file labeled 'Response to Reviewers'.
- A marked-up copy of your manuscript that highlights changes made to the original version. You should upload this as a separate file labeled 'Revised Manuscript with Track Changes'.
- An unmarked version of your revised paper without tracked changes. You should upload this as a separate file labeled 'Manuscript'.

If you would like to make changes to your financial disclosure, please include your updated statement in your cover letter. Guidelines for resubmitting your figure files are available below the reviewer comments at the end of this letter.

If applicable, we recommend that you deposit your laboratory protocols in protocols.io to enhance the reproducibility of your results. Protocols.io assigns your protocol its own identifier (DOI) so that it can be cited independently in the future. For instructions see: http://journals.plos.org/plosone/s/submission-guidelines#loc-laboratory-protocols

We look forward to receiving your revised manuscript.

Kind regards,

Farhat Afrin, Ph.D.

Academic Editor

PLOS ONE

Additional Editor Comments (if provided):

The authors need to address the issues raised by the reviewer.

[Note: HTML markup is below. Please do not edit.]

Reviewers' comments:

Reviewer's Responses to Questions

**Comments to the Author**

1. If the authors have adequately addressed your comments raised in a previous round of review and you feel that this manuscript is now acceptable for publication, you may indicate that here to bypass the “Comments to the Author” section, enter your conflict of interest statement in the “Confidential to Editor” section, and submit your "Accept" recommendation.

Reviewer #2: All comments have been addressed

Reviewer #3: (No Response)

**********

2. Is the manuscript technically sound, and do the data support the conclusions?

The manuscript must describe a technically sound piece of scientific research with data that supports the conclusions. Experiments must have been conducted rigorously, with appropriate controls, replication, and sample sizes. The conclusions must be drawn appropriately based on the data presented. 

Reviewer #2: Yes

Reviewer #3: Yes

**********

3. Has the statistical analysis been performed appropriately and rigorously? 

Reviewer #2: Yes

Reviewer #3: Yes

**********

4. Have the authors made all data underlying the findings in their manuscript fully available?

The PLOS Data policy requires authors to make all data underlying the findings described in their manuscript fully available without restriction, with rare exception (please refer to the Data Availability Statement in the manuscript PDF file). The data should be provided as part of the manuscript or its supporting information, or deposited to a public repository. For example, in addition to summary statistics, the data points behind means, medians and variance measures should be available. If there are restrictions on publicly sharing data—e.g. participant privacy or use of data from a third party—those must be specified.

Reviewer #2: Yes

Reviewer #3: Yes

**********

5. Is the manuscript presented in an intelligible fashion and written in standard English?

PLOS ONE does not copyedit accepted manuscripts, so the language in submitted articles must be clear, correct, and unambiguous. Any typographical or grammatical errors should be corrected at revision, so please note any specific errors here.

Reviewer #2: Yes

Reviewer #3: Yes

**********

6. Review Comments to the Author

Please use the space provided to explain your answers to the questions above. You may also include additional comments for the author, including concerns about dual publication, research ethics, or publication ethics. (Please upload your review as an attachment if it exceeds 20,000 characters)

Reviewer #2: None. Comments Addressed now.

Authors revised the suggestions and comments and proceed to improve and correct properly.

Reviewer #3: The overall performance is good, still, many issues should be addressed

1. Line 154:  Next, in the presence of individual patient data, single-armed observed values were converted to two-armed data to act as each other’s control group based on covariate information.

Please provide Reference for articles presented with individual data.

2. Line 162: For severity pairwise comparison, estimates of SMD served as quantitative measures of the strength of evidence against the null hypothesis of no difference in the population between mild and severe COVID-19 manifestations.

Line 165: SMD of <0.2, 0.2-0.8, and >0.8 indicated mild, moderate, and severe strength.

Line 166:  For ICU admission, survival analysis, overall effect size estimates in SMD were then converted to the odds ratio (OR) with 95%CI for better interpretation by clinical domains.

What about moderate group defined by SMD 0.2-0.8?

3. Line: 168: Decision tree to identify predictors for poor outcomes

In the manuscript, only severity was analyzed.

4. Line169:  Using laboratory features for clinical prediction, the decision tree algorithm was employed to identify the key risk factors attributed to severe COVID-19 infection.

For Risk factor? Not for cutoff value? No matter whatever it is, please provide the decision tree results as supplemental material.

5. Line 216: Ultimately, a total of 52 eligible articles were included for the quantitative synthesis of this meta-analysis study, with 52 records represented single-arm analysis, 16 records represented two-arms

Sixteen included articles have both single- and two-arms design?

And please specify the arms here, what is it?

6. Line 236:  descriptive case series, and one case-control study.

Line 212 excluded for being case records (n = 44)

What is difference between case series and case records here?

7.Table 1

1)Journal name and Publication date are not necessary to be included.

But, patients gender, age, outcomes should be provided.

8. table 2

What are the outcome measures here to predict by biological parameters?

9.Table 5

Specify the outcome “severity of COVID19”in the title.

10.Figure 2.

RSA sample size or RIS should be notified.

11.Subgroup analyses

why not conduct subgroup analyses by quality? diagnosis criteria for severity? or methods of biological parameters measurements?

12.Time point of collection of lab parameters and clinic symptoms?

**********

7. PLOS authors have the option to publish the peer review history of their article (what does this mean?). If published, this will include your full peer review and any attached files.

If you choose “no”, your identity will remain anonymous but your review may still be made public.

**Do you want your identity to be public for this peer review?** For information about this choice, including consent withdrawal, please see our Privacy Policy.

Reviewer #2: No

Reviewer #3: No

[NOTE: If reviewer comments were submitted as an attachment file, they will be attached to this email and accessible via the submission site. Please log into your account, locate the manuscript record, and check for the action link "View Attachments". If this link does not appear, there are no attachment files.]

While revising your submission, please upload your figure files to the Preflight Analysis and Conversion Engine (PACE) digital diagnostic tool, https://pacev2.apexcovantage.com/. PACE helps ensure that figures meet PLOS requirements. To use PACE, you must first register as a user. Registration is free. Then, login and navigate to the UPLOAD tab, where you will find detailed instructions on how to use the tool. If you encounter any issues or have any questions when using PACE, please email PLOS at figures@plos.org. Please note that Supporting Information files do not need this step.

# Author comment: Author response to Decision Letter 1

**DOI:** 10.1371/journal.pone.0238160.r004

25 Jul 2020

The rebuttal letter that responds to each point raised by the academic editor and the reviewer has been uploaded as a separate file labeled 'Response sheet', by the end of the manuscript as it includes some illustrations which are hard to be included here. Thanks

Attachment Submitted filename: 2-Response sheet.docx Click here for additional data file.

# Peer review: Decision Letter 2

**DOI:** 10.1371/journal.pone.0238160.r005

**Authors:** Farhat Afrin

12 Aug 2020

Diagnostic and prognostic value of hematological and immunological markers in COVID-19 infection: A meta-analysis of 6320 patients

PONE-D-20-13889R2

Dear Dr. Fawzy,

We’re pleased to inform you that your manuscript has been judged scientifically suitable for publication and will be formally accepted for publication once it meets all outstanding technical requirements.

Within one week, you’ll receive an e-mail detailing the required amendments. When these have been addressed, you’ll receive a formal acceptance letter and your manuscript will be scheduled for publication.

An invoice for payment will follow shortly after the formal acceptance. To ensure an efficient process, please log into Editorial Manager at http://www.editorialmanager.com/pone/, click the 'Update My Information' link at the top of the page, and double check that your user information is up-to-date. If you have any billing related questions, please contact our Author Billing department directly at authorbilling@plos.org.

If your institution or institutions have a press office, please notify them about your upcoming paper to help maximize its impact. If they’ll be preparing press materials, please inform our press team as soon as possible -- no later than 48 hours after receiving the formal acceptance. Your manuscript will remain under strict press embargo until 2 pm Eastern Time on the date of publication. For more information, please contact onepress@plos.org.

Kind regards,

Farhat Afrin, Ph.D.

Academic Editor

PLOS ONE

Additional Editor Comments (optional):

Reviewers' comments:

Reviewer's Responses to Questions

**Comments to the Author**

1. If the authors have adequately addressed your comments raised in a previous round of review and you feel that this manuscript is now acceptable for publication, you may indicate that here to bypass the “Comments to the Author” section, enter your conflict of interest statement in the “Confidential to Editor” section, and submit your "Accept" recommendation.

Reviewer #3: All comments have been addressed

**********

2. Is the manuscript technically sound, and do the data support the conclusions?

The manuscript must describe a technically sound piece of scientific research with data that supports the conclusions. Experiments must have been conducted rigorously, with appropriate controls, replication, and sample sizes. The conclusions must be drawn appropriately based on the data presented. 

Reviewer #3: (No Response)

**********

3. Has the statistical analysis been performed appropriately and rigorously? 

Reviewer #3: (No Response)

**********

4. Have the authors made all data underlying the findings in their manuscript fully available?

The PLOS Data policy requires authors to make all data underlying the findings described in their manuscript fully available without restriction, with rare exception (please refer to the Data Availability Statement in the manuscript PDF file). The data should be provided as part of the manuscript or its supporting information, or deposited to a public repository. For example, in addition to summary statistics, the data points behind means, medians and variance measures should be available. If there are restrictions on publicly sharing data—e.g. participant privacy or use of data from a third party—those must be specified.

Reviewer #3: (No Response)

**********

5. Is the manuscript presented in an intelligible fashion and written in standard English?

PLOS ONE does not copyedit accepted manuscripts, so the language in submitted articles must be clear, correct, and unambiguous. Any typographical or grammatical errors should be corrected at revision, so please note any specific errors here.

Reviewer #3: (No Response)

**********

6. Review Comments to the Author

Please use the space provided to explain your answers to the questions above. You may also include additional comments for the author, including concerns about dual publication, research ethics, or publication ethics. (Please upload your review as an attachment if it exceeds 20,000 characters)

Reviewer #3: (No Response)

**********

7. PLOS authors have the option to publish the peer review history of their article (what does this mean?). If published, this will include your full peer review and any attached files.

If you choose “no”, your identity will remain anonymous but your review may still be made public.

**Do you want your identity to be public for this peer review?** For information about this choice, including consent withdrawal, please see our Privacy Policy.

Reviewer #3: No

# Editor report: Acceptance letter

**DOI:** 10.1371/journal.pone.0238160.r006

**Authors:** Farhat Afrin

14 Aug 2020

PONE-D-20-13889R2 

Diagnostic and prognostic value of hematological and immunological markers in COVID-19 infection: A meta-analysis of 6320 patients 

Dear Dr. Fawzy:

I'm pleased to inform you that your manuscript has been deemed suitable for publication in PLOS ONE. Congratulations! Your manuscript is now with our production department. 

If your institution or institutions have a press office, please let them know about your upcoming paper now to help maximize its impact. If they'll be preparing press materials, please inform our press team within the next 48 hours. Your manuscript will remain under strict press embargo until 2 pm Eastern Time on the date of publication. For more information please contact onepress@plos.org.

If we can help with anything else, please email us at plosone@plos.org. 

Thank you for submitting your work to PLOS ONE and supporting open access. 

Kind regards, 

PLOS ONE Editorial Office Staff

on behalf of

Dr. Farhat Afrin 

Academic Editor

PLOS ONE
